# EM2, a Natural Product MST1/2 Kinase Activator, Suppresses Non‐Small Cell Lung Cancer via Hippo Pathway Activation

**DOI:** 10.1002/advs.202510508

**Published:** 2025-10-27

**Authors:** Siyu Yang, Huayan Xie, Qiang Lin, Liujin Zhou, Junxi Liu, Zekai Fang, Ziling Tang, Ruijie Yuan, Jinxuan Su, Sijia Li, Wenlin Wang, Mingyu Pan, Hao Wang, Xiaoyong Dai, Guocai Wang, Yubo Zhang

**Affiliations:** ^1^ Department of Pharmacology, School of Medicine, and State Key Laboratory of Bioactive Molecules and Druggability Assessment Jinan University Guangzhou 510632 China; ^2^ Department of Anesthesiology The First Affiliated Hospital Jinan University Guangzhou 510632 China; ^3^ Institute of Traditional Chinese Medicine & Natural Products, Guangdong Province Key 4 Laboratory of Pharmacodynamic Constituents of TCM and New Drugs Research, College of Pharmacy Jinan University Guangzhou 511443 China; ^4^ State Key Laboratory of Bioactive Molecules and Druggability Assessment/Guangzhou Key Laboratory of Formula‐pattern Research Center, School of Traditional Chinese Medicine Jinan University Guangzhou 510632 China; ^5^ School of Pharmacy Nanjing Medical University Nanjing China

**Keywords:** EM2, hippo signaling pathway, MST1, MST2, NSCLC

## Abstract

Lung cancer, 85% of which is non‐small cell lung cancer (NSCLC), is the cancer with the highest incidence and mortality rate worldwide. Despite recent advancements in therapeutic approaches, the efficacy of conventional radiotherapy and chemotherapy remains suboptimal, highlighting the urgent need for more effective treatment strategies. Dysregulation of kinases MST1 and MST2 (MST1/2) is implicated in the progression of NSCLC, positioning MST1/2 as a potential therapeutic target. However, no high‐selectivity and high‐efficacy MST1/2 activator is identified to date. In this study, by using computer‐aided virtual screening combined with cell experiments, EM2 is identified as a promising MST1/2‐binding candidate. Subsequent experimental validation demonstrates that EM2 significantly suppresses the proliferation, migration, and invasion of NSCLC cells by directly targeting MST1/2 and enhancing its kinase activity, thereby activating the Hippo signaling pathway and reducing nuclear translocation of the downstream effector YAP. Both in vivo xenograft models and organoid models demonstrates that EM2 effectively suppresses NSCLC tumor growth. In summary, this study not only reaffirms MST1/2 as a viable therapeutic target for NSCLC but also provides compelling experimental evidence supporting EM2 as a highly effective and promising anti‐cancer agent.

## Graphical abstract

EM2 directly binds to and activates MST1/2, enhancing their kinase activity and sequentially promoting LATS phosphorylation, which increases YAP phosphorylation. Consequently, YAP nuclear translocation is suppressed, leading to reduced transcription of downstream targets CTGF and CYR61, ultimately exerting a robust inhibitory effect on NSCLC cell proliferation, migration, and tumor growth.

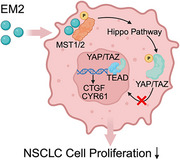

## 1 Introduction

The Sterile 20‐like serine/threonine kinases MST1 and MST2 (MST1/2) serve as essential core components of the mammalian Hippo signaling pathway, which is a highly conserved kinase cascade that is initiated by the activities of MST1/2,^[^ ^1^, ^2^ ^]^ and it plays a pivotal role in orchestrating a diverse array of physiological processes, including cell proliferation,^[^ ^3^, ^4^ ^]^ immune system regulation,^[^ ^5^, ^6^, ^7^ ^]^ development,^[^ ^8^, ^9^, ^10^, ^11^ ^]^ tissue repair,^[^ ^12^, ^13^, ^14^ ^]^ and metabolism.^[^ ^15^, ^16^ ^]^ However, aberrant expression of MST1/2 has been implicated in the pathogenesis of various diseases, with a particularly strong association with cancer.^[^ ^17^, ^18^ ^]^ Extensive research over the past decades has elucidated the tumor‐suppressive functions of MST1/2. In hepatocellular carcinoma,^[^ ^19^, ^20^, ^21^, ^22^ ^]^ colon cancer,^[^ ^23^, ^24^ ^]^ lung cancer,^[^ ^24^, ^25^ ^]^ breast cancer^[^ ^26^ ^]^ and glioma,^[^ ^27^ ^]^ downregulation of MST1/2 is associated with accelerated tumor progression. Thus, targeting MST1/2 holds significant research and clinical value, potentially bridging basic and clinical medicine to offer new cancer treatment strategies.

Lung cancer remains the most frequently diagnosed malignancy globally, with an estimated 2.5 million new cases reported annually. Moreover, it is the leading cause of cancer‐related mortality worldwide, accounting for over 1.8 million deaths per year.^[^ ^28^, ^29^ ^]^ NSCLC constitutes ≈85% of all lung cancer cases, with lung adenocarcinoma and lung squamous cell carcinoma being the predominant subtypes.^[^ ^30^, ^31^, ^32^ ^]^ Despite surgical resection and radiochemotherapy remaining crucial for achieving cure and long‐term survival in NSCLC patients, significant tumor metastasis and drug resistance have limited the 5‐year survival rate to below 20%.^[^ ^33^, ^34^, ^35^ ^]^ This underscores the urgent need to elucidate the pathogenesis of NSCLC and develop novel therapeutic strategies. Recent studies have demonstrated that MST1/2 deletion significantly promotes the development of lung cancer.^[^ ^36^, ^37^, ^38^ ^]^ Additionally, research on the traditional Chinese medicine component Neferine has shown that it can effectively inhibit lung cancer cell development by downregulating TGF‐β and modulating MST1/ROS‐induced pyroptosis.^[^ ^39^ ^]^ These findings suggest that MST1/2 holds potential as an unexplored drug target, with MST1/2 activators offering a promising avenue for NSCLC prevention.

Natural products have long been a cornerstone in the discovery and development of chemopreventive and chemotherapeutic agents, exerting a profound influence on the trajectory of anti‐cancer drug research. Currently, most of clinically utilized anti‐cancer drugs are derived from natural sources, underscoring their irreplaceable role in oncology.^[^ ^40^, ^41^, ^42^, ^43^ ^]^ In recent decades, plant‐based medicines have emerged as a prominent research hotspot and focal point in the realm of anti‐tumor therapeutics. EM‐2, a low‐molecular constituent isolated from *Elephantopus mollis H.B.K*., exerts potent antineoplastic activity against human hepatocellular carcinoma by specifically suppressing autophagic flux.^[^ ^44^ ^]^ Subsequent investigations have revealed a marked synergistic interaction between EM‐2 and epirubicin in both in vitro and in vivo, resulting in enhanced cytotoxicity and tumor growth inhibition.^[^ ^45^ ^]^ These findings position EM‐2 as a promising agent for cancer therapy.

In the present study, we meticulously assembled an extensive library of natural compounds and drugs. Leveraging virtual screening technology and a series of rigorous experimental validations, we successfully confirmed that compound EM2 can specifically target MST1/2. By enhancing the activity of MST1/2 kinase to modulate the Hippo signaling pathway, EM2 effectively inhibits the proliferation of NSCLC, while simultaneously inducing apoptosis and senescence in these cancer cells. Moreover, the results of the xenograft models and organoid model demonstrated that EM2 significantly promotes the demise of NSCLC cells and robustly activates the Hippo signaling pathway. Based on these comprehensive research findings, we have compelling evidence to assert that EM2 is a novel MST1/2 kinase activator with significant therapeutic potential for NSCLC.

## 2 Results

### 2.1 EM2 Directly Targets MST1/2

**Figure 1:**
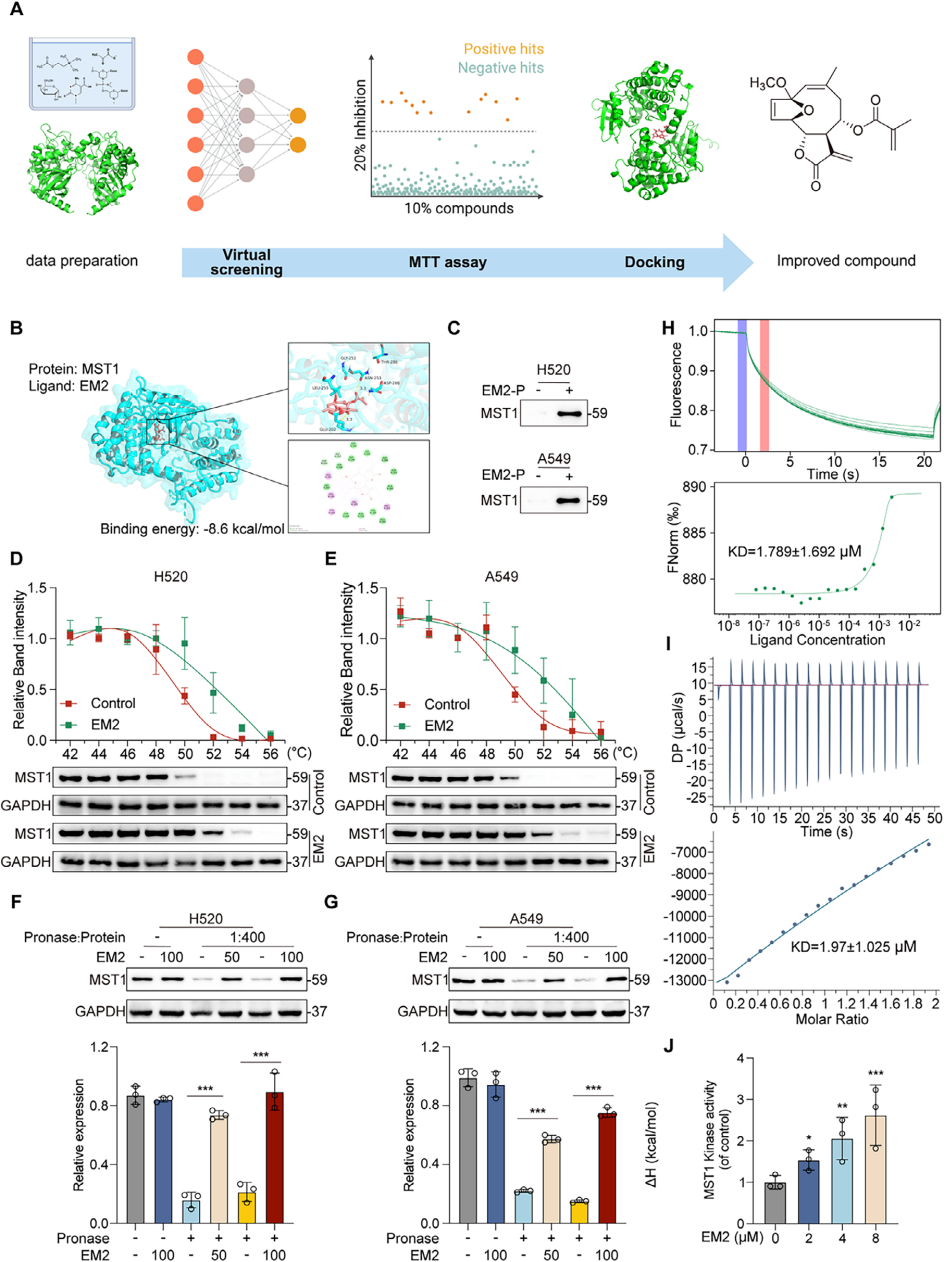
The binding ability of EM2 and MST1/2. A) Workflow of drug screening. B) Molecular docking of EM2 with MST1. C) Pull‐down followed by immunoblotting to verify EM2 directly targeting to MST1 proteins in situ. D,E) The binding of EM2 to MST1 was determined by CETSA approach in H520 D) and A549 E) cells. F,G) The binding of EM2 to MST1 was determined by DARTS approach in H520 F) and A549 G) cells. H) The binding affinity between EM2 and MST1 was determined using microscale thermophoresis assay. I) The binding affinity between EM2 and MST1 was determined using ITC. J) The kinase activity of MST1 after treatment with different concentrations of EM2. Data are presented as mean ± SD. *p < 0.05, **p < 0.01, ***p < 0.001.

In order to identify novel MST1/2 activators, we employed virtual artificial intelligence screening on a natural compound library comprising 1120 natural products. The top 10% of compounds (10 µM) with the highest potential were subjected to MTT assays to evaluate cell viability. Among these, compounds exhibiting cytotoxic effects were further subjected to refined molecular docking studies and in vitro kinase activity experiments by ADP‐Glo assay kits. Ultimately, EM2 emerged as the optimal candidate (**Figure** **1**A,B; Figure S3A,B, Table S1, Supporting Information). Molecular docking analysis revealed the presence of Glu202, Gly252, Asn253, Leu255, Asp266, and Thr286 in the binding pocket of MST1 (Figure 1B). Molecular dynamics simulations show that the EM2‐MST1 complex has strong dynamic stability (Figure S3C–G, Supporting Information). In addition, co‐incubation with excess EM2 could compete with the labeling of EM2‐P in situ in A549 cells (Figure S3H–J, Supporting Information). And a pull‐down experiment was conducted to verify the direct interaction between EM2 and MST1 (Figure 1C). CETSA demonstrated that EM2 enhanced MST1 thermal stability, reducing its temperature‐dependent degradation (Figure 1D,E). DARTS assays further showed EM2‐mediated protection of MST1 from proteolysis. MST1 in the control group was almost completely digested by Pronase, resulting in a pronounced loss of signal. In contrast, MST1 pre‐incubated with EM2 formed a drug‐protein complex that remained largely intact after protease treatment, yielding a substantially stronger band at the expected molecular weight (Figure 1F,G). Microscale thermophoresis confirmed dose‐dependent binding affinity (Figure 1H). Similarly, ITC showed a binding KD of 1.97 ± 1.025 µM for EM2 and MST1 consistent with high affinity (Figure 1I). More importantly, MST1/2 functions through its kinase activity, which significantly increased with EM2 concentration (Figure 1J). These results establish that EM2 directly binds to MST1/2 with high affinity.

### 2.2 EM2 Suppresses Proliferation, Migration, and Invasion of NSCLC Cells

**Figure 2:**
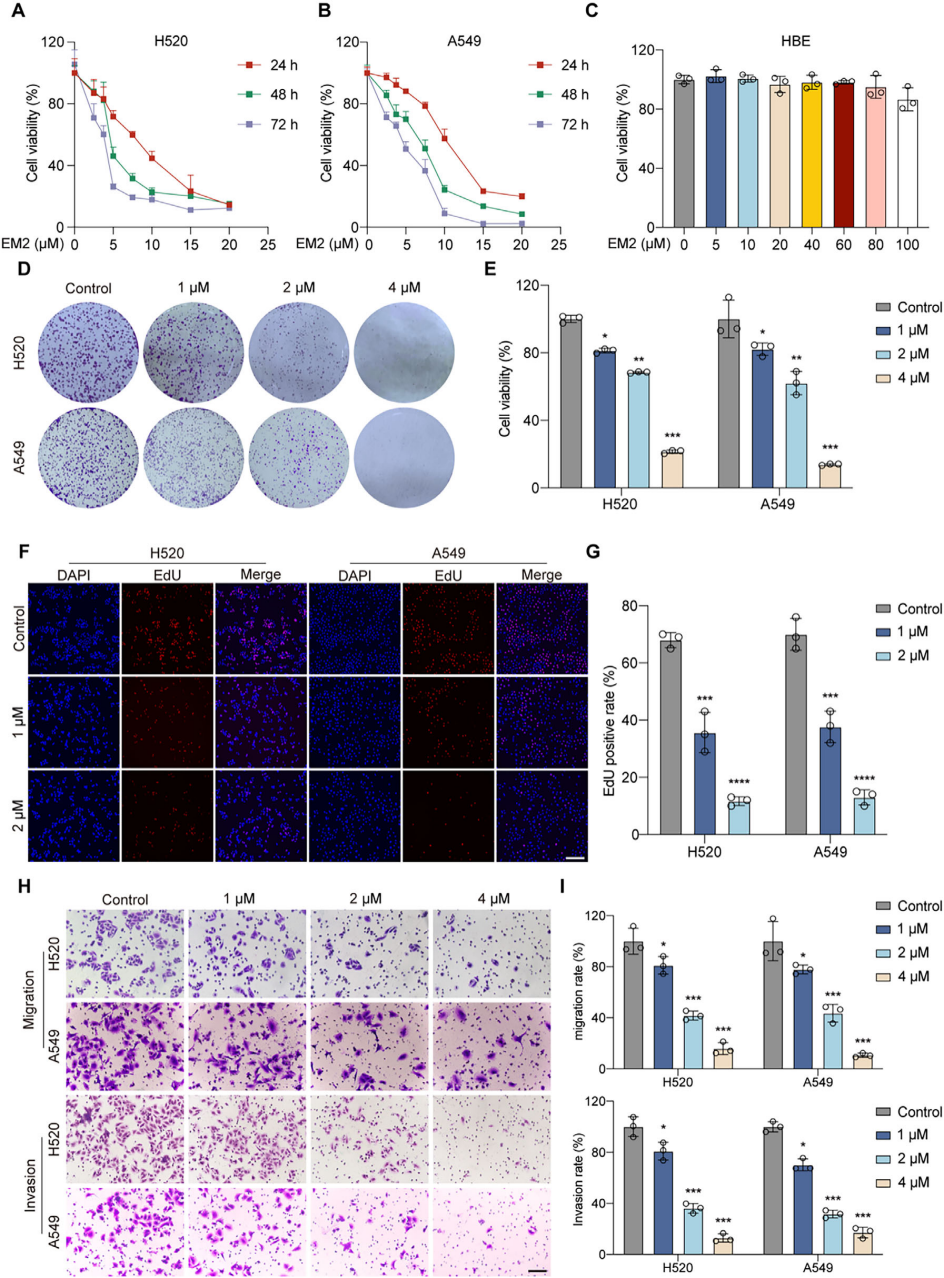
The effect of EM2 on proliferation, migration, and invasion of NSCLC cells. A,B) Effects of EM2 on the viability of H520 A) and A549 B) cells were determined by the MTT method after treatment for 24, 48, and 72 h, respectively. C) Effects of EM2 on the viability of HBE cells were determined by the MTT method after treatment for 24 h. D,E) Representative colony formation results D) and quantitative analysis E) of cells after EM2 treatment of H520 and A549. F,G) Representative images F) and quantification G) of the effect of EM2 on DNA synthesis in H520 and A549 cells. H,I) Representative images H) and quantification I) of effects of EM2 on migration and invasion in H520 and A549 cells. Following treatment with EM2, the cells were subjected to the Transwell migration and invasion assays. Data are presented as mean ± SD. *p < 0.05, **p < 0.01, ***p < 0.001.

To evaluate the anti‐tumor effect of EM2, cells were treated with different concentrations of EM2 for 24, 48, and 72 h, respectively, and cell viability was determined by the MTT assay. EM2 inhibits cell proliferation in a dose‐and time‐dependent manner (**Figure** **2**A,B; Figure S4A, Supporting Information). However, EM2 is non‐toxic to normal cell lines HBE (Figure 2C) and HUVEC (Figure S4B, Supporting Information). Colony formation assay (Figure 2D,E) and EdU assay (Figure 2F,G) further confirmed that EM2 inhibits the proliferation of NSCLC cells. In view of the influence of cell migration and invasion on the therapeutic effect and prognosis of lung cancer, we evaluated it's in vitro inhibitory effect on the motility of NSCLC cells through the transwell assay. Following EM2 treatment, both H520 and A549 cells exhibited a significant reduction in migratory and invasive capabilities compared to control group (Figure 2H,I; Figure S4C, Supporting Information). These results indicate that EM2 effectively suppresses NSCLC cell proliferation, invasion, and invasiveness in vitro.

### 2.3 EM2 induces Cell Cycle Arrest at G1 Phase and Cellular Senescence in NSCLC Cells

**Figure 3:**
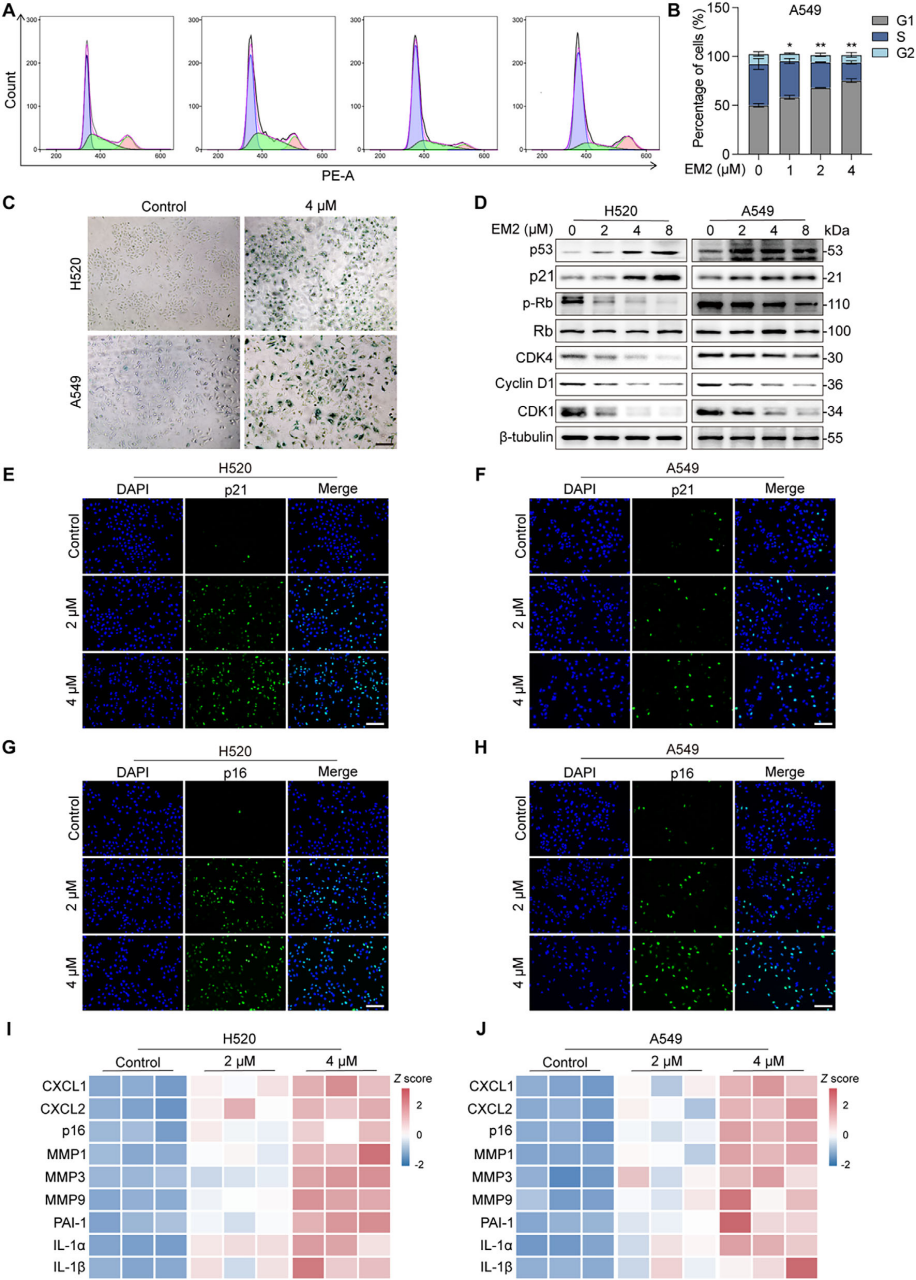
The influence of EM2 on the cell cycle and senescence of NSCLC cells. A,B) Representative images A) and quantification B) of cell cycle distribution measured by flow cytometry in EM2‐treated A549 cells. C) SA‐β‐gal staining analysis in EM2‐treated H520 and A549 cells. D) Immunblot analysis of p53, p21, p‐RB, RB, CDK4, CDK6, Cyclin D1, and CDK1 in H520 and A549 cells after treatment with EM2. E,F) IF analysis of p21 in EM2‐treated H520 E) and A549 F) cells. G,H) IF analysis of p16 in EM2‐treated H520 G) and A549 H) cells. I,J) Heatmap showing the expression of indicated genes in EM2‐treated H520 I) and A549 J) cells. Data are presented as mean ± SD. *p < 0.05, **p < 0.01, ***p < 0.001.

To further explore the mechanism by which EM2 inhibits NSCLC cells, we further conducted flow cytometry analysis on the cell cycle and found that the cells were significantly blocked in the G1 phase after EM2 treatment (**Figure** **3**A,B). Given that cell cycle arrest accompanies cellular senescence, SA‐β‐Gal staining verified that after EM2 treatment, SA‑β‑Gal–positive cells accumulated (Figure 3C). Western blotting revealed downregulation of Cyclin D1 and CDK4 treated with EM2, suggesting that EM2 may inhibit cell proliferation by blocking the G1/S phase. Furthermore, EM2 dose‐dependently increased protein levels of p53, p21 and p16, consistent with senescent chromatin remodeling (Figure 3D–H). Moreover, senescence is accompanied by senescence‐associated secretory phenotype (SASP), secreting soluble signaling factors (interleukins, chemokines, and growth factors) into intercellular context. Gene expressions of *IL‐1α, IL‐1β, IL‐6, MMP1, MMP3, MMP9, TNF‐α, CXCL2*, and *PAI‐1* all significantly enriched after EM2 treatment (Figure 3I,J). Together, these results demonstrated that EM2 induces cell cycle arrest at G1 phase and cellular senescence in NSCLC cells.

### 2.4 EM2 Activates the Hippo Pathway in NSCLC Cells

**Figure 4:**
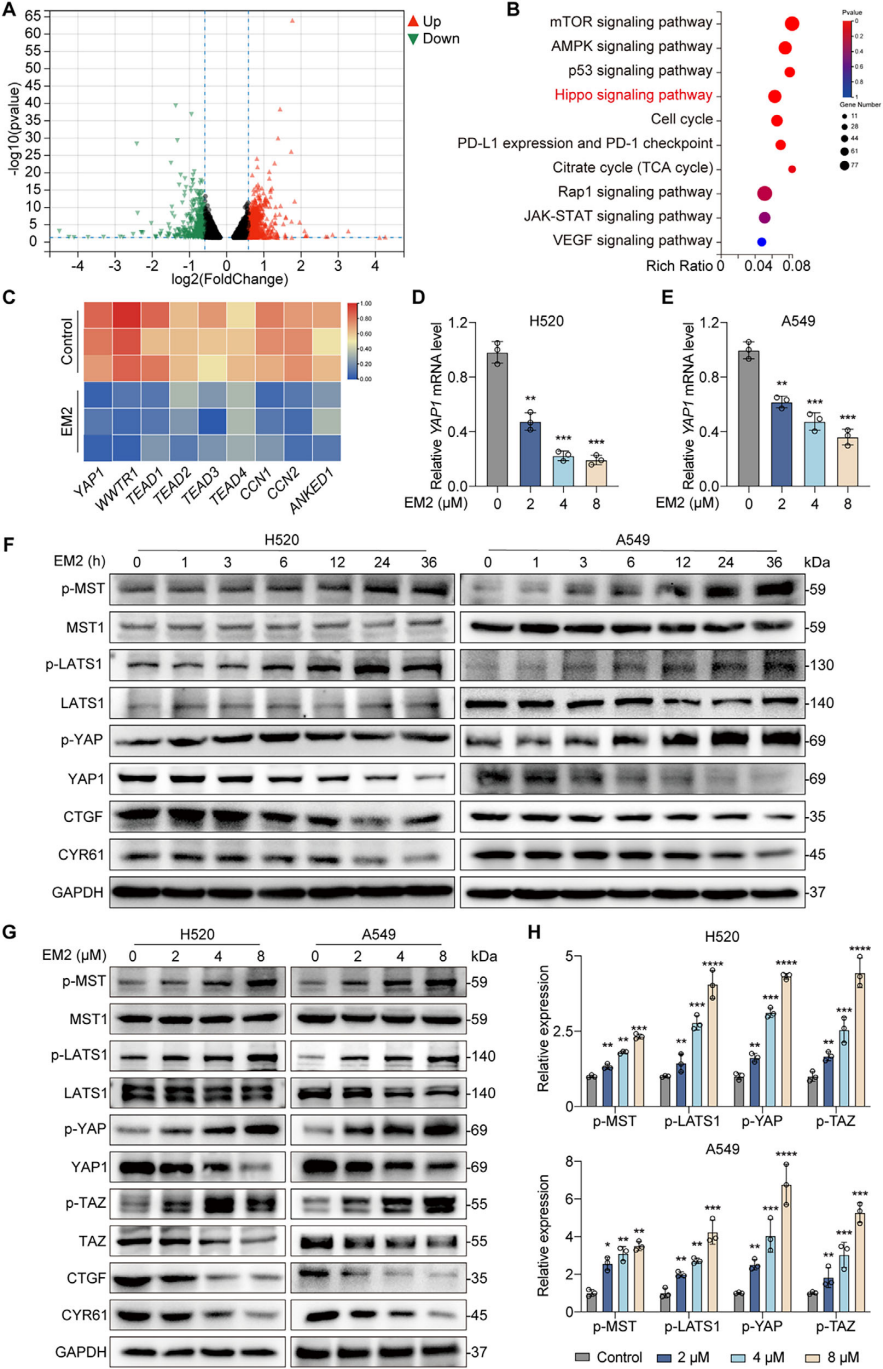
Hippo signaling pathway involved in EM2‐induced NSCLC. A) A549 cells were treated with EM2 for RNA sequencing, and a volcano plot of differentially expressed genes was generated by analysis (up‐regulated genes are shown in red; down‐regulated genes are shown in blue; non‐regulated genes are shown in gray) B) KEGG pathway enrichment analysis of differentially expressed genes. C) Clustering heat map of downstream genes of the Hippo pathway by RNA sequencing. D,E) YAP mRNA levels were measured by RT‐qPCR in EM2‐treated H520 D) and A549 E) cells. F) Cells were treated with 8 µM EM2 for 0, 1, 3, 6, 12, 24, and 36 h and Western blotting was performed to analyze the expression levels of Hippo pathway proteins. G) Cells were treated with the specified concentration of EM2, and Western blotting was performed to analyze the expression levels of hippo pathway proteins. H) Quantification of the protein levels indicated in G). Data are presented as mean ± SD. *p < 0.05, **p < 0.01, ***p < 0.001.

To elucidate the molecular mechanisms underlying the anti‐NSCLC effects of EM2, RNA sequencing was performed in A549 cells treated with or without EM2. Compared to the control group, EM2 treatment led to the upregulation of 3145 genes and the downregulation of 2535 genes (**Figure** **4**A). KEGG enrichment analysis of differentially expressed genes revealed significant enrichment of the Hippo signaling pathway (Figure 4B). RNA‐seq data reveals a widespread downregulation of transcript levels for core components of the Hippo signaling pathway (Figure 4C). Further, EM2 suppresses YAP mRNA expression in a dose‐dependent manner (Figure 4D,E). Moreover, EM2 treatment increased phosphorylation of MST, LATS, YAP, and TAZ over time and dose, without altering total MST or LATS levels. Concurrently, protein and mRNA level of YAP, TAZ, CTGF and CYR61 decreased in a dose‐dependent manner (Figure 4F–H; Figure S5A–E, Supporting Information). Collectively, these findings suggest that EM2 activates the Hippo signaling pathway in NSCLC cells.

### 2.5 EM2 Exhibits Anticancer Effects Dependent on YAP Activity

**Figure 5:**
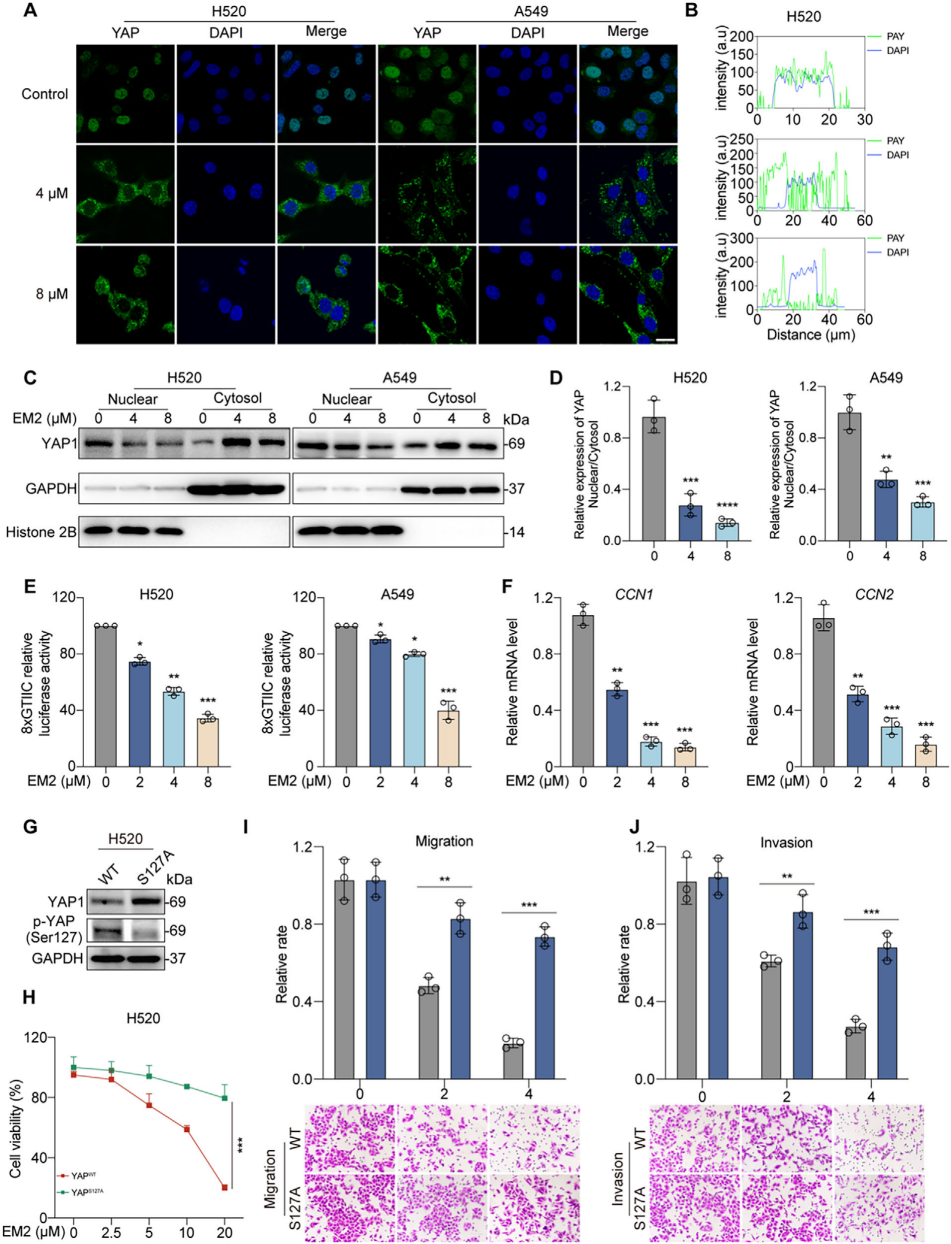
The role of YAP activity in EM2 anti‐NSCLC. A,B) After EM2 treatment with H520 and A549, representative images A) and quantification B) of YAP localization were detected by IF staining. C,D) Cytoplasmic and nuclear distributions of YAP in H520 and A549 cells treated with or without EM2 for 12 h. Histone 2B and GAPDH were used as endogenous references for nuclear and cytosolic fractions C). The indicated protein expression levels were quantified D). E) Transcriptional activity of YAP/TEAD complex in H520 and A549 cells. Cells were transfected with the reporter plasmids and treated with EM2 at the indicated concentrations for 24 h. F) mRNA levels of C*CN1* and *CCN2* in H520 cells treated with EM2. RT‐qPCR data were normalized to GAPDH levels and presented as fold‐change compared with control cells. G) Western blotting was used to detect the expressions of YAP and p‐YAP in YAP WT and YAP S127A H520 cells. H) Cell viability of A549 and H520 cells transfected with YAP WT and YAP S127A were treated with 0, 2.5, 5, 10, and 20 µM EM2 for 24 h, and the cell viability was detected by MTT. I,J) Representative images and quantification of EM2 on the migration I) and invasion J) in H520 cells transfected with YAP WT and YAP S127A. Following treatment with indicated concentrations of EM2, the cells were subjected to Transwell migration and invasion assays. Data are presented as mean ± SD. *p < 0.05, **p < 0.01, ***p < 0.001.

YAP, as a key effector of the Hippo pathway, mainly exerts its transcriptional regulatory function through translocation within the nucleus. To further investigate the effect of EM2 on the subcellular localization of YAP, the results of IF staining showed that YAP was mainly retained in the cytoplasm after EM2 treatment (**Figure** **5**A,B; Figure S6A, Supporting Information). Further protein isolation analysis indicated that EM2 significantly reduced the level of YAP protein in the nucleus and simultaneously increased the expression of YAP in the cytoplasm (Figure 5C,D; Figure S6B, Supporting Information). To explore the functional effect of EM2 on the transcriptional activity of YAP, we transfected an 8× GTIIC‐Luciferase reporter plasmid containing a TEAD‐binding enhancer sequence, which could activate the YAP/TEAD complex. Data show that EM2 treatment significantly inhibits YAP/TEAD‐dependent transcriptional activity in a dose‐dependent manner (Figure 5E; Figure S6C, Supporting Information). Furthermore, EM2 treatment led to a significant decrease in the expression of mRNA of the downstream genes *CCN1* and *CCN2* of YAP (Figure 5F; Figure S6D, Supporting Information).

To further explore the dependence of EM2's anti‐NSCLC effect on YAP activity, we constructed a constitutive active YAP S127A mutant plasmid (Figure 5G; Figure S6E, Supporting Information), which led to the continuous transcriptional activation of YAP. The results showed that the inhibitory effect of EM2 on cell proliferation (Figure 5H; Figure S6F, Supporting Information), migration (Figure 5I; Figure S6G, Supporting Information), and invasion (Figure 5J; Figure S6H, Supporting Information) in the YAP S127 group was significantly weakened compared with the YAP WT group. These findings further indicate that the anti‐tumor effect of EM2 largely depends on YAP phosphorylation and the prevention of its nuclear translocation, thereby inhibiting YAP‐mediated transcriptional activation and cell proliferation and movement.

### 2.6 MST1/2 Inhibition Reverses EM2's Antitumor Effects In Vitro

**Figure 6:**
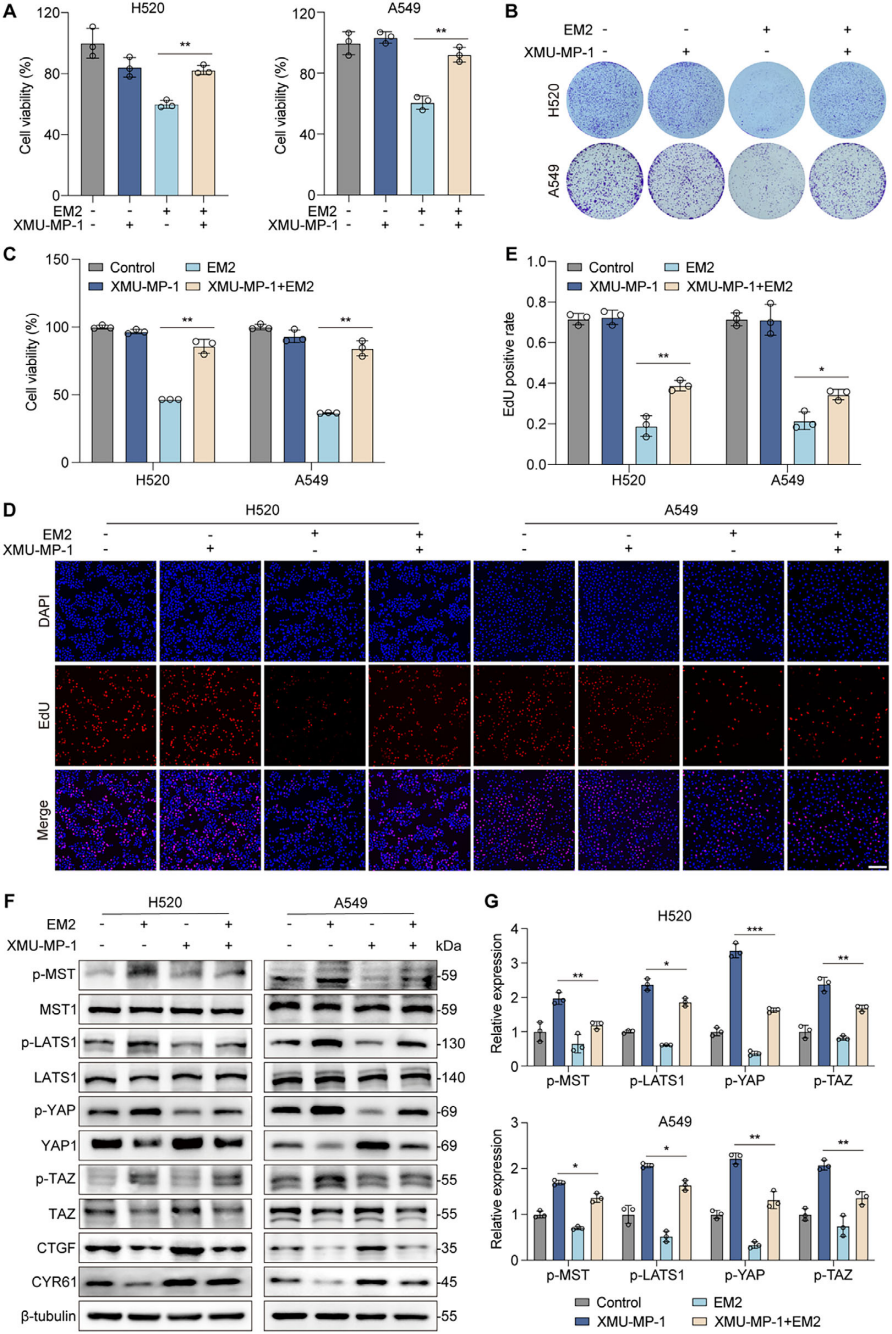
The anti‐tumor activity of EM2 was counteracted by MST1/2 inhibitor in vitro. A) Cell viability of H520 and A549 cells treated by EM2 (8 µM) with or without XMU‐MP‐1 (3 µM). B,C) Representative images B) and quantification C) of colony formation assay of H520 and A549 cells treated by EM2 (2 µM) with or without XMU‐MP‐1 (0.5 µM). D) EdU assay of H520 and A549 cells treated by EM2 (2 µM) with or without XMU‐MP‐1 (0.5 µM). E) Quantification of Edu positive in H520 and A549 cells. F) Immunoblot analysis of p‐MST, p‐LATS1/2, p‐YAP, and p‐TAZ in H520 and A549 cells treated by EM2 (8 µM) with or without XMU‐MP‐1 (3 µM). β‐tubulin served as the loading control. G) Quantification of the protein levels indicated in H520 and A549 cells. Data are presented as mean ± SD. *p < 0.05, **p < 0.01, ***p < 0.001.

To further verify that the effect of EM2 depends on the activation of MST1/2, we jointly treated NSCLC cells with XMU‐MP‐1 (an MST1/2 kinase inhibitor) and EM2. XMU‐MP‐1 significantly rescued cell viability (**Figure** **6**A) and colony formation (Figure 6B,C) suppressed by EM2, and restored EdU positive cell rate (Figure 6D,E). We simultaneously conducted functional experiments using MST1 knockdown cells and obtained the same results (Figure S7A–G, Supporting Information). Moreover, XMU‐MP‐1 attenuated EM2‐induced Hippo pathway activation, demonstrating that EM2's in vitro anticancer effects require MST1/2 activity (Figure 6F,G). Collectively, these data proves that EM2 mediates the activation of the Hippo signaling pathway through MST1/2, thereby exerting an anti‐tumor effect.

### 2.7 EM2 Suppresses Tumor Growth via MST1/2‑Mediated Hippo Signaling In Vivo

**Figure 7:**
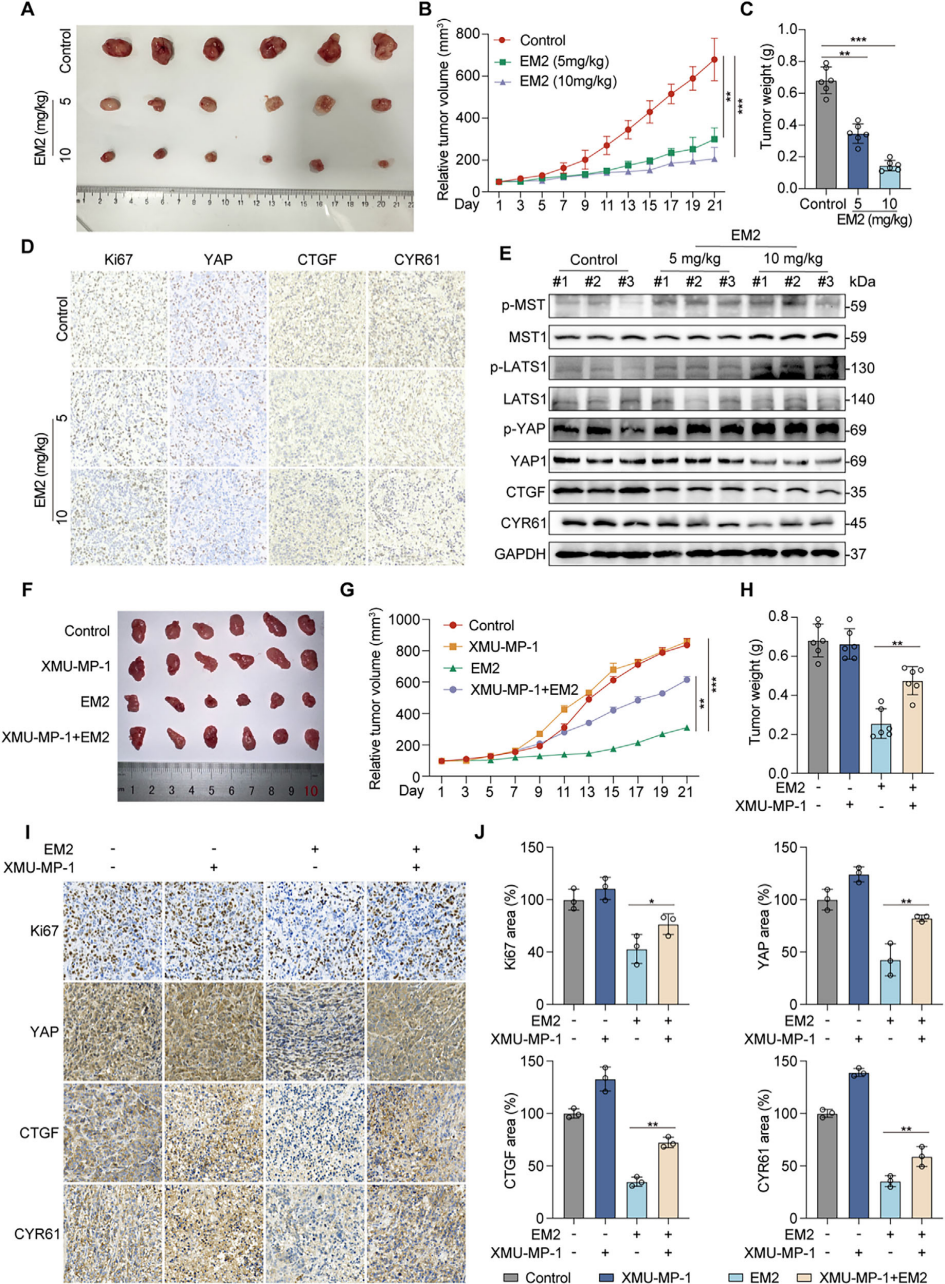
The role and mechanism of EM2 in inhibiting the tumor growth of NSCLC in vivo. Representative images of the subcutaneous tumor model in mice of control and EM2 (5 or 10 mg kg^−1^) group. B) Tumor volume in control and EM2 (5 or 10 mg kg^−1^) group. C) Quantification and analysis of the tumor weight in control and EM2 (5 or 10 mg kg^−1^) group. D) The expression levels of Ki67, YAP, CTGF, and CYR61 in tumors assayed by IHC in control and EM2 (5 or 10 mg kg^−1^) group. E) Expression levels of MST1, LATS1, and YAP and their phosphorylation forms along with downstream protein CTGF and CYR61 were examined by Western blotting in control and EM2 (5 or 10 mg kg^−1^) group. F) Representative images of tumors in subcutaneous mouse model, grouped by treatment with EM2 with or without XMU‐MP‐1. G) Tumor volume in each group of EM2 with or without XMU‐MP‐1. H) Quantification and analysis of the tumor weight in each group of EM2 with or without XMU‐MP‐1. I,J) Representative images I) and quantification J) of the expression levels of Ki67, YAP, CTGF, and CYR61 in tumors assayed by IHC in each group of EM2 with or without XMU‐MP‐1. Data are presented as mean ± SD. *p < 0.05, **p < 0.01, ***p < 0.001.

To further verify the anti‐tumor effect of EM2 in vivo, a subcutaneous xenograft tumor model was established. Results showed that EM2 significantly inhibit subcutaneous transplant tumors growth (**Figure** **7**A–C). Body weight and histological analysis of major organs (heart, liver, spleen, lung, and kidney) showed no obvious histological damage or morphological changes in the EM2‐treated group (Figure S8A–C, Supporting Information), indicating good safety of EM2 in vivo. Further, IHC showed that compared with the control group, the EM2‐treated group significantly inhibited the expression of Ki67, YAP, CTGF, and CYR61 (Figure 7D). Similarly, immunoblotting results showed that EM2 activate the Hippo signaling pathway in vivo, upregulating p‐MST, p‐LATS, and p‐YAP levels, and downregulating CTGF, CYR61 simultaneously (Figure 7E).

To determine the dependency of MST1/2 in EM2‐induced blockade of NSCLC progression, a subcutaneous xenograft tumor model and the MST1/2 inhibitor XMU‐MP‐1 were employed. The inhibition of EM2 in the growth of subcutaneous transplanted tumors was reversed by XMU‐MP‐1 (Figure 7F–H). Similarly, there was no significant difference in the body weights of mice (Figure S8B,D, Supporting Information). Furthermore, the IHC results showed that, compared with the control group, XMU‐MP‐1 could reverse the expressions of Ki67, YAP, CTGF, and CYR61 after EM2 treatment (Figure 7I,J). Together, the above data confirmed that EM2 could suppress tumor growth via MST1/2‑mediated Hippo signaling in vivo.

### 2.8 EM2 Inhibits Growth Of Patient‑Derived NSCLC Organoids

**Figure 8:**
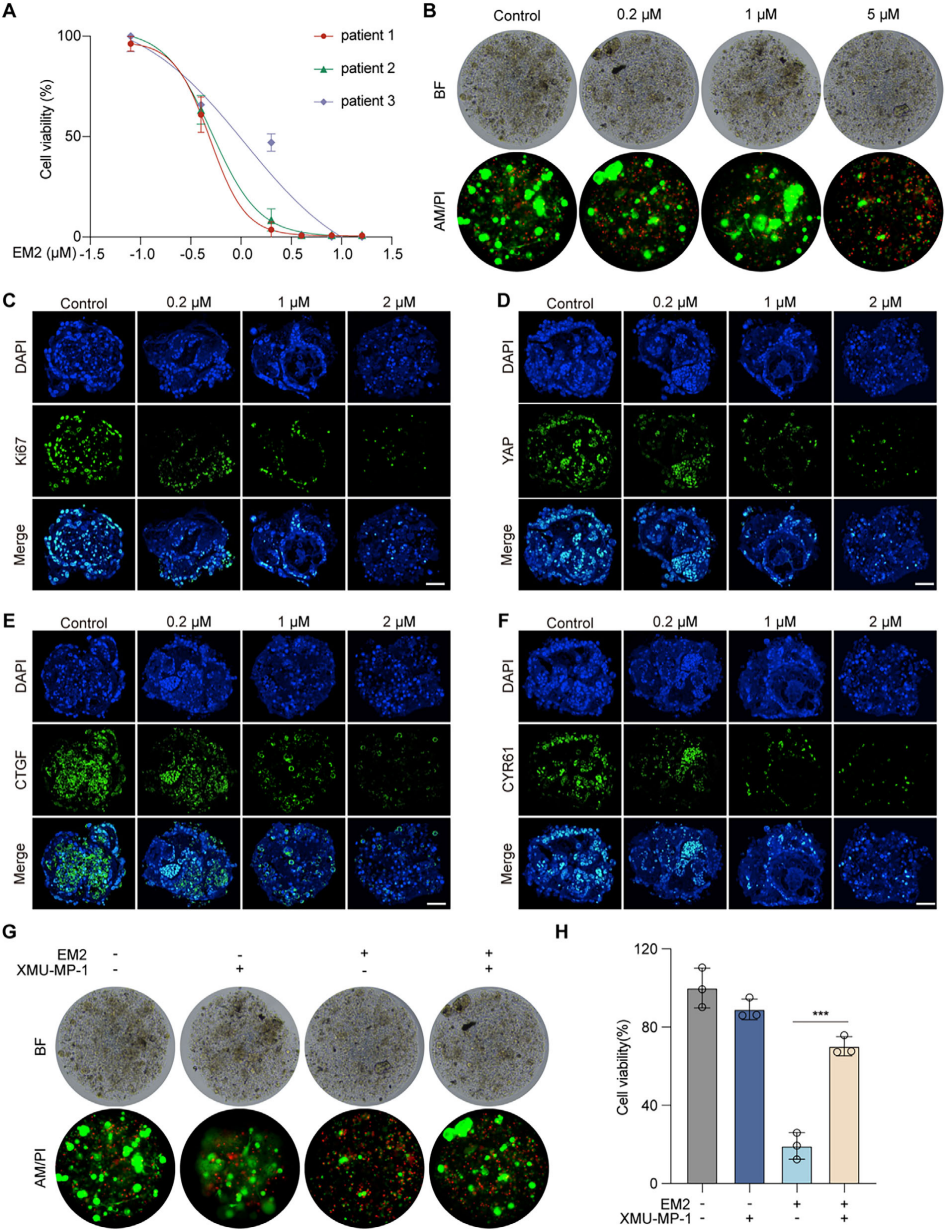
The effect of EM2 on organoids of non‐small cell lung cancer. A) The effect of EM2 on the organoid viability of NSCLC. B) Representative images of the viability of NSCLC organoids treated with different concentrations of EM2. C) Representative IF images of EM2 on the expression of Ki67 in NSCLC organoids. D) IF representative image of EM2 on the expression of YAP in organoids of NSCLC. E) IF representative images of EM2 on the expression of CTGF in NSCLC organoids. F) IF representative images of EM2 on the expression of CYR61 in NSCLC organoids. G,H) Representative images G) and quantification H) of cell viability of NSCLC organoids treated by EM2 with or without XMU‐MP‐1.

Organoids have become an important tool for human experimental research due to their ability to highly simulate the tissue structure and function of real organs. We generated tumor organoids from three NSCLC patient samples and confirmed their viability by calcein AM/PI staining. Treatment with EM2 yielded IC_50_ values ranging from 0.54 to 1.15 µM, demonstrating potent anti‑tumor activity (**Figure** **8**A,B). IF revealed that EM2 reduced expression of Ki‑67 (Figure 8C), YAP (Figure 8D) and its downstream targets CTGF (Figure 8E) and CYR61 (Figure 8F). In addition, XMU‐MP‐1 significantly saved the organoid death caused by EM2 (Figure 8G,H). These findings validate EM2's potent efficacy in patient‑derived organoids.

## 3 Discussion

The management of NSCLC is complicated by several key challenges. The tumor's high heterogeneity significantly increases treatment complexity. Additionally, the escalating drug resistance of tumor cells to conventional chemotherapeutics severely limits therapeutic efficacy. Moreover, the high toxicity of current treatment regimens often causes severe side effects, which not only impair patients' quality of life but also reduce treatment tolerance.^[^ ^35^, ^46^ ^]^ Against this backdrop, natural plant extracts have emerged as a promising alternative for anti‐cancer drug development. Unlike traditional chemotherapies, these extracts exhibit robust biological activities with lower toxicity and fewer side effects, offering distinct advantages and broad potential for application in cancer treatment.

*Elephantopus tomentosus H.B.K*., a member of the Elephantopus family, is a traditional Chinese herbal medicine known for its properties of clearing heat, detoxifying, promoting diuresis, reducing fever, and possessing anti‐inflammatory effects.^[^ ^47^, ^48^ ^]^ Recent studies have demonstrated that this plant is rich in bioactive chemical components, particularly sesquiterpene lactones and triterpenoids, which exhibit potent anti‐cancer activities by inhibiting tumor growth.^[^ ^49^ ^]^ Notably, molephantinin, a compound isolated from *Elephantopus tomentosus H.B.K*. in the 1980s, has been shown to exert significant anti‐leukemia effects against P‐388 lymphocytic leukemia, Ehrlich carcinoma, and Walker 256 cancer.^[^ ^50^ ^]^ Another compound, EM23, has been reported to induce excessive accumulation of ROS and inhibit the proliferation of K562 and HL‐60 cells by suppressing the TNF‐α/NF‐κB signaling pathway.^[^ ^51^ ^]^ As a sesquiterpene lactone, EM2 has also been found to inhibit hepatocellular carcinoma proliferation^[^ ^44^ ^]^ and enhance the sensitivity of breast cancer cells to epirubicin.^[^ ^45^ ^]^ Despite these findings, the effects of EM2 and its extracts on NSCLC have not been previously explored. In this study, we report that EM2 significantly inhibits the proliferation of NSCLC cells in cell, animal and organoid models. Moreover, EM2 is non‐toxic to normal cell lines and animals. These findings provide novel insights into potential therapeutic targets and strategies for the treatment of NSCLC, highlighting the promising anticancer potential of EM2.

The Hippo signaling pathway is a highly conserved kinase cascade composed of MST1/2, LATS1/2, YAP. Given its critical role in regulating cell proliferation, apoptosis, senescence, and the tumor microenvironment, the Hippo pathway has garnered significant attention in recent years.^[^ ^1^, ^52^, ^53^, ^54^ ^]^ Dysregulation of the Hippo pathway, particularly low expression or functional loss of its core kinases MST1/2 and LATS1/2, leads to aberrant activation of downstream effectors YAP. This, in turn, promotes the development and progression of NSCLC and various other malignancies.^[^ ^55^ ^]^ Due to the pivotal role of the Hippo signaling pathway in NSCLC, therapeutic strategies targeting this pathway have emerged as a major research focus. MST1/2, as a key upstream regulator of the Hippo pathway, is considered a critical factor in the pathogenesis and progression of NSCLC. While most current strategies target the downstream YAP/TAZ‐TEAD complexes, these approaches are limited by their impact on terminal effector.^[^ ^56^, ^57^, ^58^ ^]^ In contrast, research on Hippo pathway activators remains in its infancy. Recent studies have identified several compounds that modulate the Hippo pathway. For instance, Irbelsartan effectively inhibits chemotherapy resistance by suppressing the Hippo/YAP1/c‐Jun/stemness/iron metabolism axis.^[^ ^59^ ^]^ Gracillin suppresses cancer progression by enhancing the interaction between Merlin and LATS, thereby activating the Hippo signaling pathway.^[^ ^60^ ^]^ However, small molecule drugs with high selectivity targeting MST1/2 are rarely reported.

In this study, we used virtual screening, MTT, molecular docking, pull‐down, CESTA, DARTS, ITC and microscale thermophoresis experiments to clarify the direct targeting of MST1/2 by EM2. Mechanistically, EM2 enhances the kinase activity of MST1/2 and increases its phosphorylation to activate the Hippo signaling pathway, resulting in the down‐regulation of oncofactors such as CTGF and CYR61, thereby inhibiting the proliferation of NSCLC cells and inducing cell senescence. Thus, this study not only provides novel insights into the activation mechanisms of the Hippo signaling pathway but also offers robust experimental evidence supporting EM2 as a potential activator of this pathway and a promising candidate for cancer therapy.

Despite providing compelling evidence for the therapeutic efficacy of EM2 in NSCLC, our study is subject to several limitations. First, while the beneficial effects of EM2 on NSCLC have been demonstrated, its pharmacokinetic profile in animal models remains unclear. Additionally, the precise binding sites of EM2 on MST1/2 require further validation. Notably, we did not assess the comparative efficacy of EM2 versus established YAP inhibitors.

In conclusion, this study has successfully identified compound EM2 as an activator of MST1/2. Our findings demonstrate that EM2 exerts robust inhibitory effects on the proliferation of NSCLC cells, thereby highlighting its potential as an anti‐NSCLC therapeutic agent. Given these results, EM2 emerges as a highly promising candidate for the development of novel NSCLC therapies and warrants further investigation and development.

## 4 Conclusion

**Figure 9:**
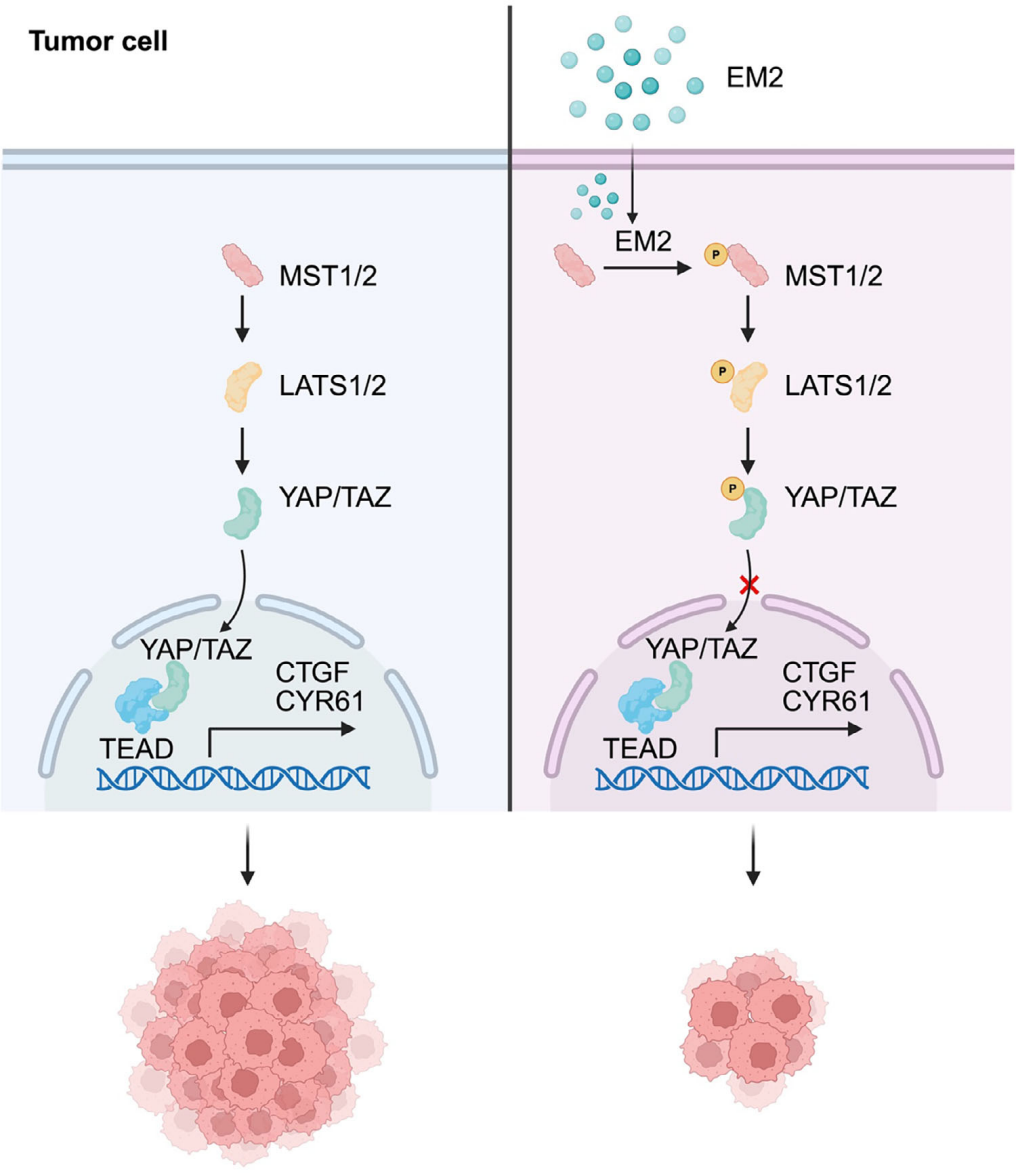
Schematic illustration of EM2‐induced suppression of cancer through targeting MST1/2 and activation of the Hippo signaling pathway. EM2 directly targets MST1/2, enhancing its kinase activity to promote LATS and YAP phosphorylation. This cascade reduces YAP nuclear translocation and diminishes CTGF and CYR61 mRNA translation, thereby exerting a potent inhibitory effect on NSCLC progression.

EM2 was identified as an effective MST1/2 agonist through virtual screening, MTT activity evaluation, molecular docking and MST1/2 kinase activity. By activating the Hippo signaling axis and blocking the nuclear translocation of YAP, EM2 significantly inhibits the proliferation, migration, invasion and tumor growth of NSCLC in cell, organoid and xenograft models, demonstrating excellent development potential (**Figure** **9**).

## 5 Experimental Section

### AI‐Driven Drug Screening

The method is as shown in the previous articles.^[^ ^61^, ^62^ ^]^ This study first takes the BindingDB dataset as the core training dataset for drug‐target interaction(DTI)prediction, laying a data foundation for subsequent model construction and performance optimization. To screen potential candidate compounds targeting MST1, the trained DTI prediction model was applied to the database of Traditional Chinese Medicine of Jinan University for virtual screening. In the model training and feature processing stage, the training and validation of the Transformer model were completed first. For the input data, the RDKit Python chemoinformatics toolkit was used to preprocess the drug molecular structure, and atomic‐level features at the molecular level were obtained through steps such as atomic topology analysis and chemical bond feature extraction. At the same time, the MST1 protein sequence was standardized, and the above‐mentioned molecular features and protein sequence features were uniformly converted into feature embedding vectors compatible with the input format of the Transformer model to ensure that the model can efficiently learn the association patterns between features. Ultimately, based on the combined score of the compounds output by the model and the prediction of MST1, the candidate compounds screened out were ranked in descending order, providing a priority reference for subsequent experimental verification.

### Chemicals

The EM2 powder was extracted and purified independently by our research group. The structures of these compounds were elucidated using a comprehensive suite of spectroscopic analyses, including mass spectrometry, ultraviolet‐visible spectroscopy, infrared spectroscopy, and nuclear magnetic resonance, in conjunction with their chemical properties (Figure S1, Supporting Information).

### Antibodies

Antibodies against MST1 (#3682), LATS1 (#3477), YAP (#12395), YAP/TAZ (#8418), phospho‐MST1 (Thr183)/MST2 (Thr180) (#49332), phospho‐LATS1 (Ser909) (#9157), phospho‐YAP (Ser127) (#13008), phospho‐TAZ (Ser89) (#59971), phospho‐Mob1 (Thr35) (#8699), CTGF (#86641), CYR61 (#14479) Phospho‐Rb(Ser780) (#2808), CDK4 (#12790), CDK6 (#3136), and Cyclin D1 (#55506) were purchased from Cell Signaling Technology. Antibody against GAPDH (10494‐1‐AP) and β‐Tubulin (80713‐1‐RR) was purchased from Proteintech. β‐Actin (T0022) and Ki67 (#AF0198) were purchased from Affinity. p21 (F0170) were purchased from Selleck, respectively.

### Molecular Docking

The crystal structure of MST1/2 was retrieved from the Research Collaboratory for Structural Bioinformatics Protein Data Bank (http://www.rcsb.org/pdb/). File form transformation was conducted by Open Babel software. Molecular docking was performed using Autodock Vina 1.1.2, with semi‐flexible docking adopted. In this method, the receptor was considered rigid while the ligand was flexible. PyMOL version 2.2.0 was utilized for visualization.

### Cell Thermal Stability (CETSA)

Cells were resuspended in PBS with protease and phosphatase inhibitors, separated into fractions, and heated at a gradient of 42, 44, 46, 48, 50, 52, 54, and 56 °C for 3 min using a PCR thermal cycler. After heating, samples were exposed to two freeze‐thaw cycles using liquid nitrogen. Following centrifugation at 12,000 rpm for 20 min, the supernatant was collected, boiled, and subjected to SDS‐PAGE.

### Drug Affinity Response Target Stability (DARTS)

A549 and H520 cells were treated with either DMSO or EM2 at a concentration of 50 and 100 µm for a duration of 3 h. Cell lysates were harvested at room temperature, and then subjected to a 15 min incubation with an equivalent volume of Pronase (0.3 µg mL^−1^). Subsequently, protease inhibitors were added to the samples. The expression of MST1 in all samples was analyzed using SDS‐PAGE.

### Pull Down

To investigate whether MST1/2 is the target of EM2, we synthesized the EM2‐P probe (Figure S3B, Supporting Information)and performed pull‐down experiments. Cells were cultured to 80–90% confluence, treated with the probe (with or without competitors) for 4 h, and then lysed with RIPA buffer. After measuring protein concentration by BCA assay, the lysates were subjected to a click reaction at room temperature for 2 h. Proteins were precipitated with acetone overnight, collected by centrifugation, and dissolved in 1.5% SDS‐PBS. The supernatant was incubated with streptavidin beads at 4 °C overnight, washed, and the bound proteins were eluted and analyzed by SDS‐PAGE.

### Microscale Thermophoresis

A549 cells were transfected with the MST1‐GFP plasmid, and then the protein lysates were collected with RIPA lysis buffer. Subsequently, EM2 was continuously diluted with PBS. Then treat the protein lysate with EM2 solution for 5 min. The obtained mixture was loaded into capillaries and MST measurements were performed using the Monolith NT.115 instrument (Nanotemper, Munich, Germany).

### Isothermal Titration Calorimetry (ITC)

ITC measurements were carried out utilizing a MicroCal PEAQ‐ITC instrument (Malvern). The calorimetric investigations were conducted at a temperature of 25 °C in a buffer comprising 50 mM HEPES at pH 7.5, 150 mM NaCl, and 2 mM DTT. EM2, housed in the titration syringe, was incrementally introduced into the reaction chamber containing the MST1 protein, with each injection separated by an interval of 120 s and the stirring velocity set to 750 rpm min^−1^. The thermodynamic response was monitored using high‐feedback mode, and the raw calorimetric data were processed with the MicroCal PEAQ‐ITC analysis software suite.

### Kinase Activity In Vitro

MST1/2 kinase activity was measured using an ADP‐Glo kinase assay kit (V6930, Promega, USA) according to the manufacturer's instructions. Briefly, kinase assays were performed in white solid 96‐well plates with kinase buffer containing active MST1, MOB1a, ATP, DMSO or the indicated concentrations of EM2 at 25 °C for 30 min. Then, the ADP‐Glo reagent was added and samples were incubated at 25 °C for 40 min. The resulting luminescence signal was measured using a fluorescence microplate reader. The descriptions were added accordingly.

### Animal Study

Female BALB/c nude mice (5–6 weeks, 18–20 g) were purchased from Beijing Vital River Laboratory Animal Technology Co., Ltd., and were maintained according to Jinan University's Policy on the Care and Use of Laboratory Animals (Approval No. 20240514‐01). All experiments were approved by the Institutional Animal Care and Use Committee of Jinan University. For xenograft tumor model, A549 cells (5 × 10^6^ cells in 100 µL of PBS) were injected subcutaneously into the right axilla of the mice. When tumors reached 100 mm^3^, mice were divided into three groups: EM2 (5 or 10 mg kg^−1^) and control, administered daily via intraperitoneal injection. Tumor volume (V) was measured every two days using the formula: V = length × width^2^ × 0.5. After 21 days, mice were euthanized, and major organs were fixed and paraffin‐embedded for H&E staining. Tumors were photographed, weighed, and stored at −80 °C for further analysis.

### NSCLC Organoid Culture

Human studies followed international ethical guidelines (Approval No. LL‐KY‐2024047‐02). Lung cancer tissues were collected from consenting patients undergoing tumor resection, with normal tissues also removed. Cancer cells were isolated via mechanical disruption and enzymatic digestion, then suspended in low growth factor matrix gel (Corning) at 2.0 × 10⁷ cells mL^−1^. Organoids were cultured in advanced DMEM/F12 medium containing 100 ng mL^−1^ EGF, 50 ng mL^−1^ bFGF, 100 ng mL^−1^ noggin, 10% R‐spondin‐1 conditioned medium, 1.25 mM N‐acetyl cysteine, 10 mM nicotinamide, and 50 nM A83‐01. Microscopic observation showed successful organoid formation with increasing tumor sphere size over time.

### Statistical Analysis

Data were obtained from at least three independent experiments and presented as mean ± SD or SEM. Prior to analysis, data were examined for normality and homogeneity of variance. Statistical analyses were performed using GraphPad Prism 8.4.0. Group differences were assessed by Student's t‐test or one‐way ANOVA, with comparisons to untreated controls unless stated otherwise. Overall survival was compared using the Kaplan‐Meier method and log‐rank test. *p* value of <0.05 was considered significant, with ^*^ *p* < 0.05, ^**^ *p* < 0.01, and ^***^ *p* < 0.001 indicating significance levels.

## Conflict of Interest

The authors declare no conflict of interest.

## Supporting information



Supporting Information

## Data Availability

The data that support the findings of this study are available from the corresponding author upon reasonable request.
