# Supplementary material for: EM2, a Natural Product MST1/2 Kinase Activator, Suppresses Non‐Small Cell Lung Cancer via Hippo Pathway Activation
Source: Adv Sci (Weinh). 2025 Oct 27;13(1):e10508. doi: 10.1002/advs.202510508 (PMC12767113; doi:10.1002/advs.202510508)
Supplement: Supplementary file 1 — Supporting Information [file ADVS-13-e10508-s001.docx]

**Appendix A. Supplementary data**

**Table S1** Compound docking score with MST1.

| Number | Binding energy |
| --- | --- |
| EM-8 | -8.1 |
| EM-19 | -7.8 |
| EM-4 | -7.8 |
| EM-11 | -8.8 |
| EM-2 | -8.6 |
| JST-2 | -8.4 |
| EM-12 | -7.9 |
| DC-19 | -8.3 |
| WP-45 | -7.4 |
| EM-15 | -8.9 |
| N7 | -8.7 |
| EM(Y)-5 | -8.5 |
| EM-7 | -8.1 |
| EM-3 | -8.3 |
| JST-4 | -8.0 |
| EM-1 | -7.5 |
| WP-5 | -8.0 |
| BM-43 | -7.8 |
| HYZ-4 | -8.4 |
| CL-15 | -8.1 |
| DAR-33 | -8.6 |

**Table S2** sequence of qPCR primers.

| Primer name | Base sequence (5' to3') |
| --- | --- |
| GAPDH-F | CAATGACCCCTTCATTGACC |
| GAPDH-R | GACAAGCTTCCCGTTCTCAG |
| YAP1-F | TCCACCAGTGCAGCAGAATA |
| YAP1-R | TTGGGTCTAGCCAAGAGGTG |
| CTGF-F | CCTGCAGGCTAGAGAAGCA |
| CTGF-R | TGGAGATTTTGGGAGTACGG |
| CYR61-F | ACCAATGACAACCCTGAGTG |
| CYR61-R | AAACATCCAGCGTAAGTAAACC |
| IL-1α-F | TGTATGTGACTGCCCAAGATGAAG |
| IL-1α-R | AGAGGAGGTTGGTCTCACTACC |
| IL-1β-F | CCAGCTACGAATCTCCGACC |
| IL-1β-R | TCGTGCACATAAGCCTCGTT |
| MMP1-F | ATGTGGAGTGCCTGATGTGG |
| MMP1-R | TTGTCCCGATGATCTCCCCT |
| MMP3-F | ACTCACAGACCTGACTCGGTT |
| MMP3-R | AAGCAGGATCACAGTTGGCTGG |
| MMP9-F | GCCACTACTGTGCCTTTGAGTC |
| MMP9-R | CCCTCAGAGAATCGCCAGTACT |
| p16-F | CCAACGCACCGAATAGTTACG |
| p16-R | GCGCTGCCCATCATCATG |
| CXCL1-F | ACCCAAACCGAAGTCATAGC |
| CXCL1-R | TCTCCGTTACTTGGGGACAC |
| CXCL2-F | AACTGCGCTGCCAGTGCT |
| CXCL2-R | CCCATTCTTGAGTGTGGCTA |
| PAI-1-F | CTCATCAGCCACTGGAAAGGCA |
| PAI-1-R | GACTCGTGAAGTCAGCCTGAAAC |

**Table S3 MST1 siRNA sequence**

|  | Sense strand (5’-3’) | Antisense strand (5’-3’) |
| --- | --- | --- |
| SiRNA NC | UUCUCCGAACGUGUCACGUTT | ACGUGACACGUUCGGAGAATT |
| siMST#1 | GAUUGUUGCUAUUAAGCAATT | UUGCUUAAUAGCAACAAUCTT |
| siMST#2 | GCUACAAUAUUACAAUCAATT | UUGCUUAAUAGCAACAAUCTT |
| siMST#3 | GGACCAGGACGAUGAAGAATT | UUCUUCAUCGUCCUGGUCCTT |


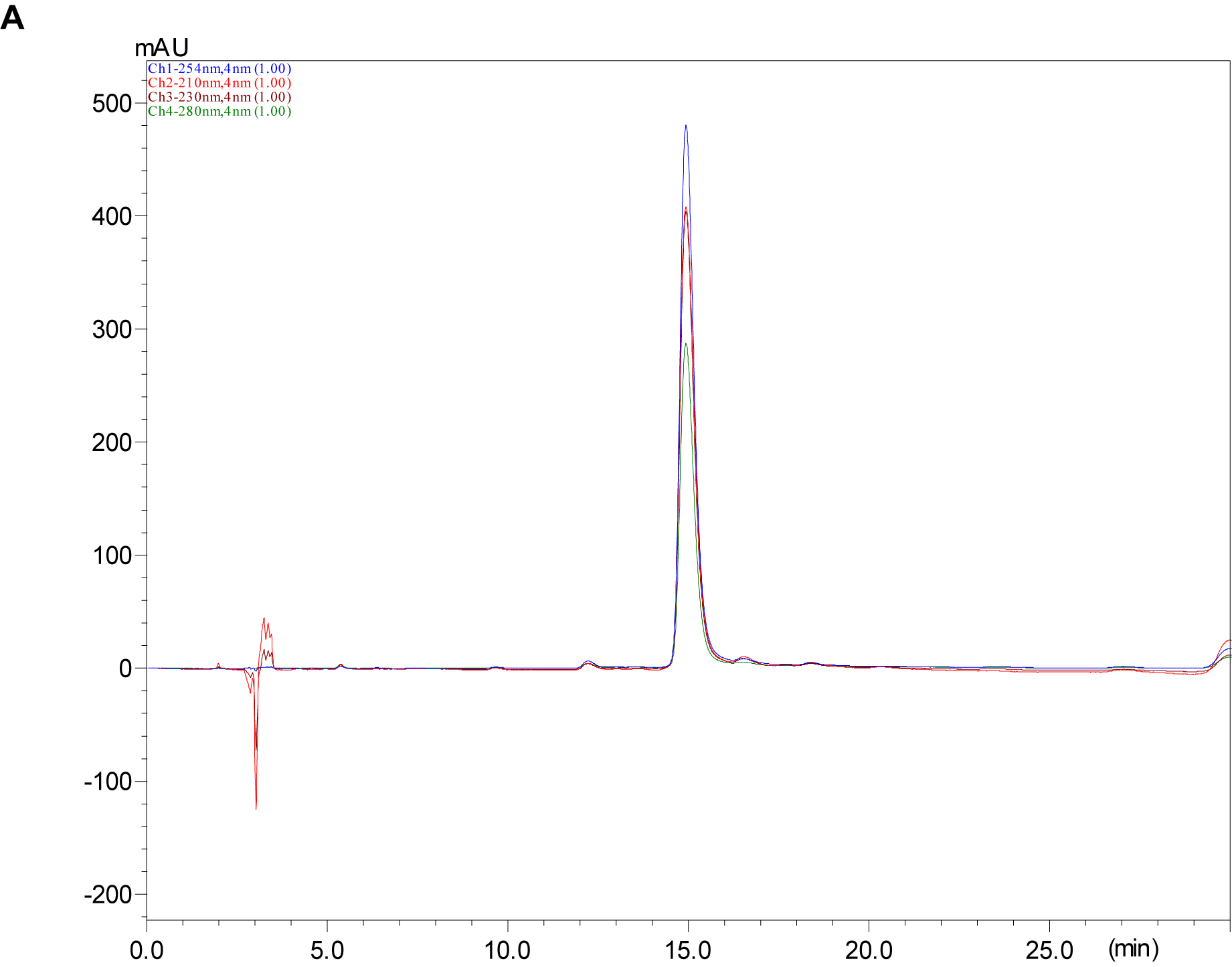


**Figure 1 The chromatogram of EM-2.**

**
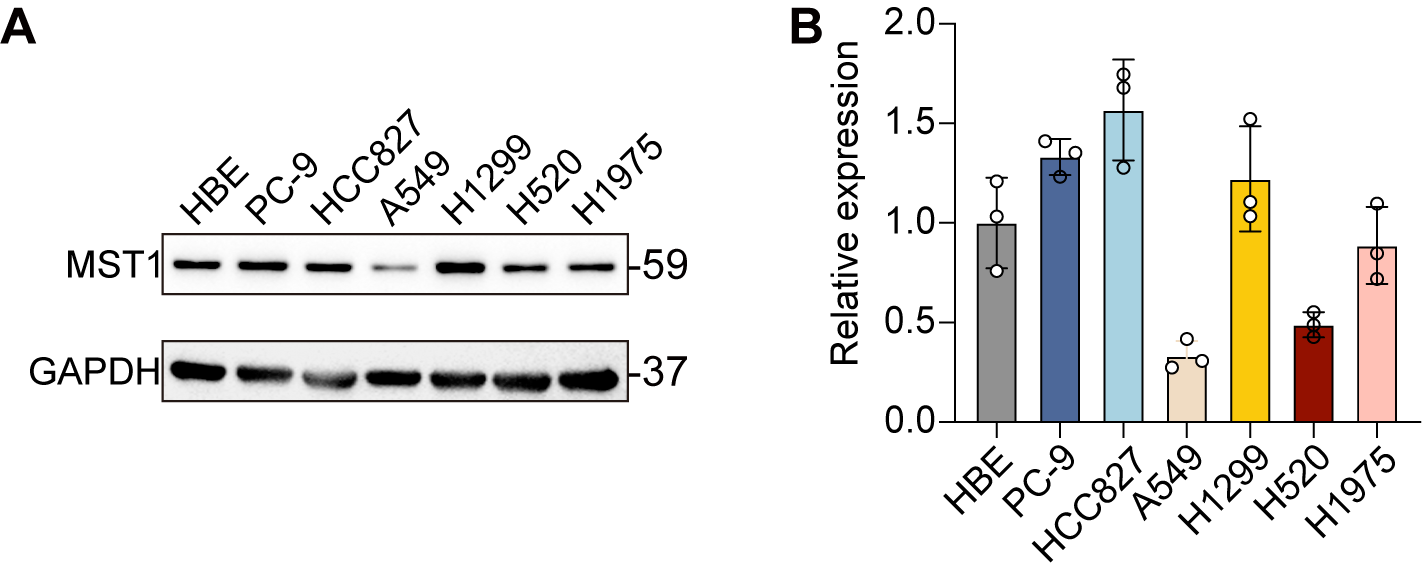
**

**Figure S2 The protein level of MST1.**

(A) The protein level of MST1 was examined in six lung cancer cell lines (PC-9, HCC827, A549, H1299, H1975, H520) and one human bronchial epithelial cell line (HBE). MST1 was highly expressed in HBE cells, whereas it was lost or expressed at low levels in A549, H1975, and H520 cells. (B) Quantification of Figure A.


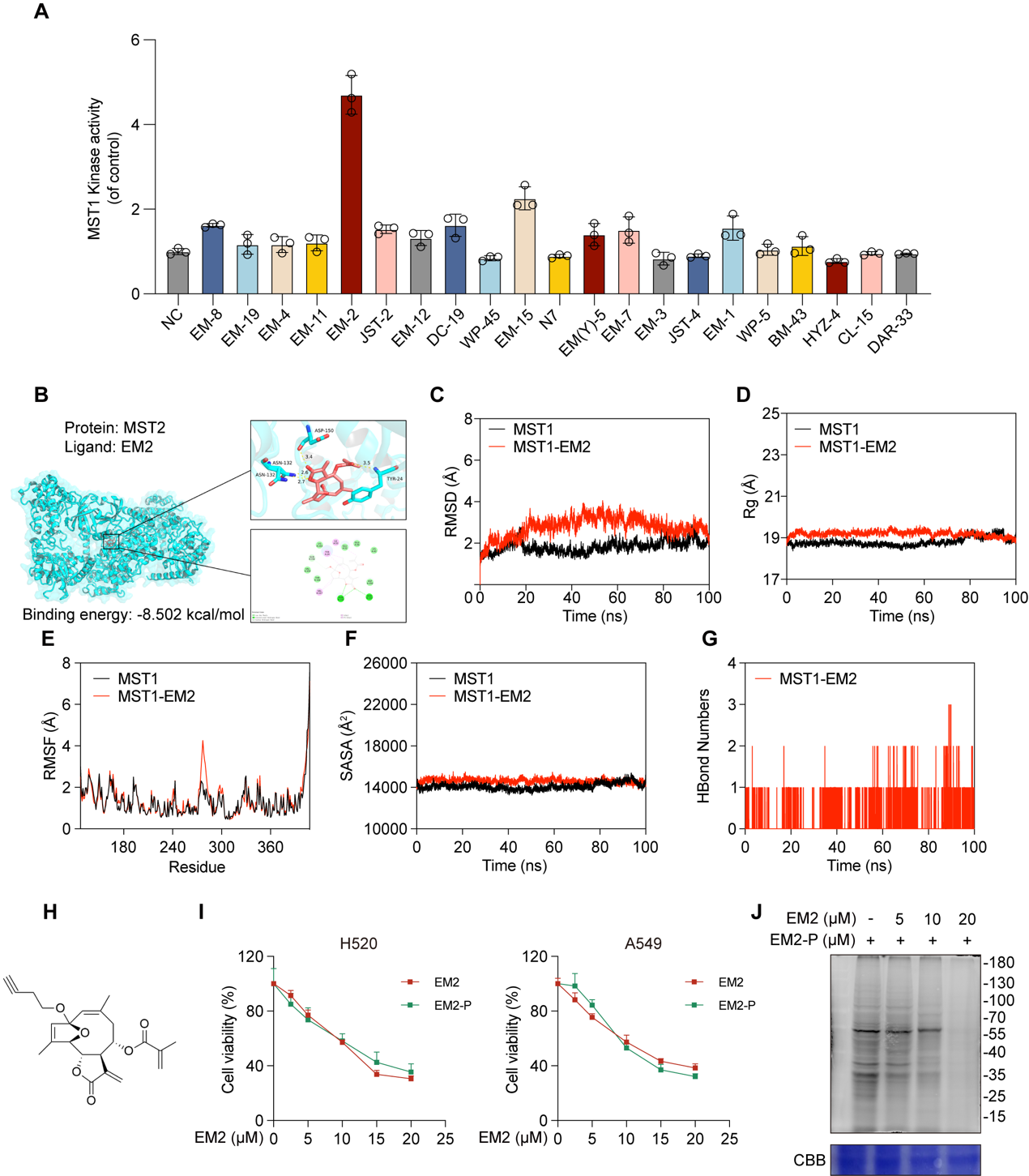


**Figure S3 The binding ability of EM2 and MST1/2.**

(A) The activation effect of candidate compounds (10 µM) on MST1 enzyme activity was evaluated in vitro by using the ADP-Glo analysis method. (B) Molecular docking of EM2 with MST2. (C) Root mean square deviation (RMSD) of the complexes. (D) Radius of gyration (Rg) of the complexes.  (E) Root mean square fluctuation (RMSF) of the complexes. (F) Solvent accessible surface area (SASA) of the complexes.  (G) Number of hydrogen bonds formed by the complex (H-Bond). (H) The structure of EM2-P. (I) Effects of EM2 and EM2-P on the viability of H520 and A549 cells were determined by the MTT method after treatment for 24 h, respectively. (J) The competition of in situ protein labeling with EM2-P by EM2 in A549 cells.


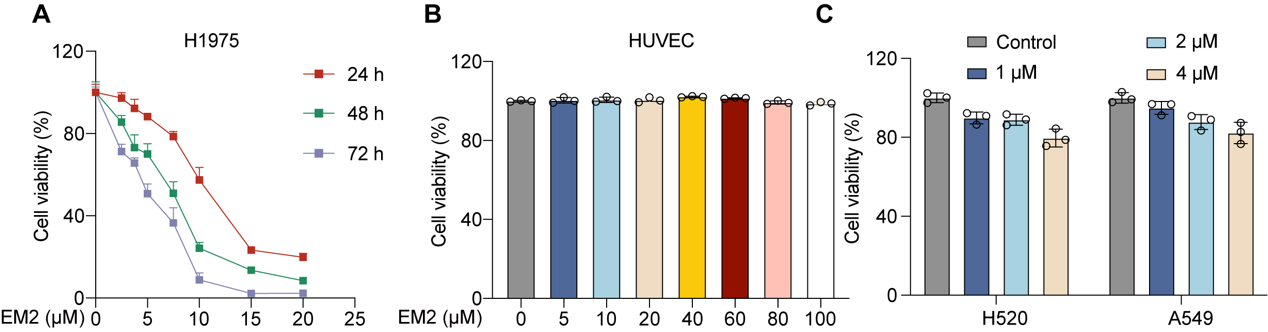


**Figure S4 EM2 suppresses proliferation of NSCLC cells, and shows no cytotoxicity in HUVEC line.**

(A) Time-dependent effect of EM2 on in H1975 cell viability, assessed by MTT assay after 24, 48, 72 h of treatment. (B) Dose-dependent effect of EM2 on in and HUVEC cell viability, assessed by MTT assay after 24 h of treatment. (C) After 24 h of treatment, the effects of 1, 2 and 4 μM EM2 on the viability of H520 and A549 cells were determined by the MTT method.


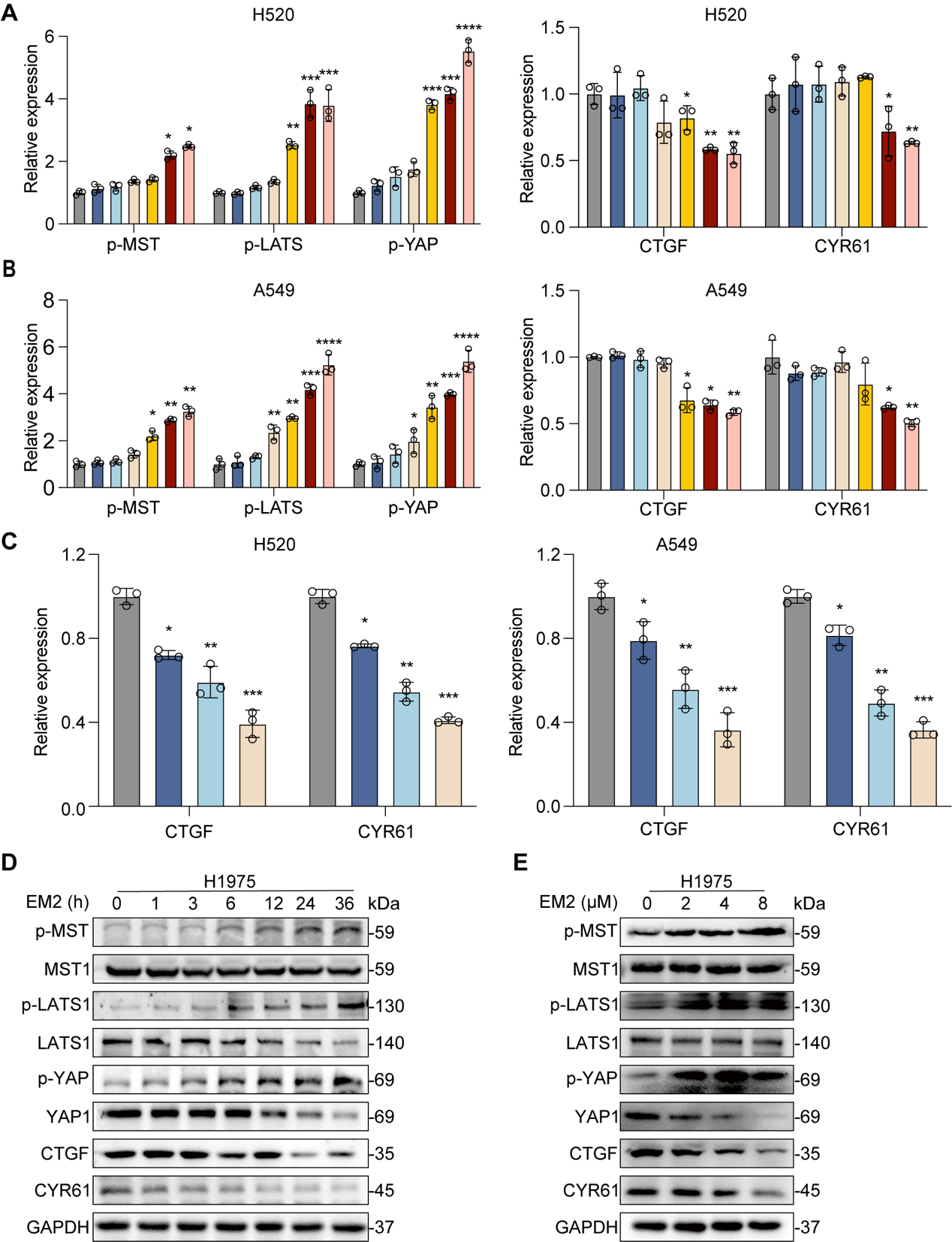


**Figure S5 EM2 activates the Hippo signaling pathway in H1975 cells.**

(A-B) Quantitative EM2 activates the expression of Hippo pathway proteins in H520 (A) and A549 (B) cells in a time-dependent manner. (C) Quantitative EM2 reduces the expression of CTGF and CYR61 proteins in H520 and a549 cells in a dose-dependent manner. (D) EM2 activates the Hippo signaling in a time-dependent manner in H1975 cells. Cells were treated with EM2 for 0, 1, 3, 6, 12, 24 and 36 h and subjected to Western blotting analysis. (E) EM2 activates the Hippo signaling in a dose-dependent manner in cancer cells. Cells were treated with EM2 at indicated concentrations and subjected to Western blotting analysis.


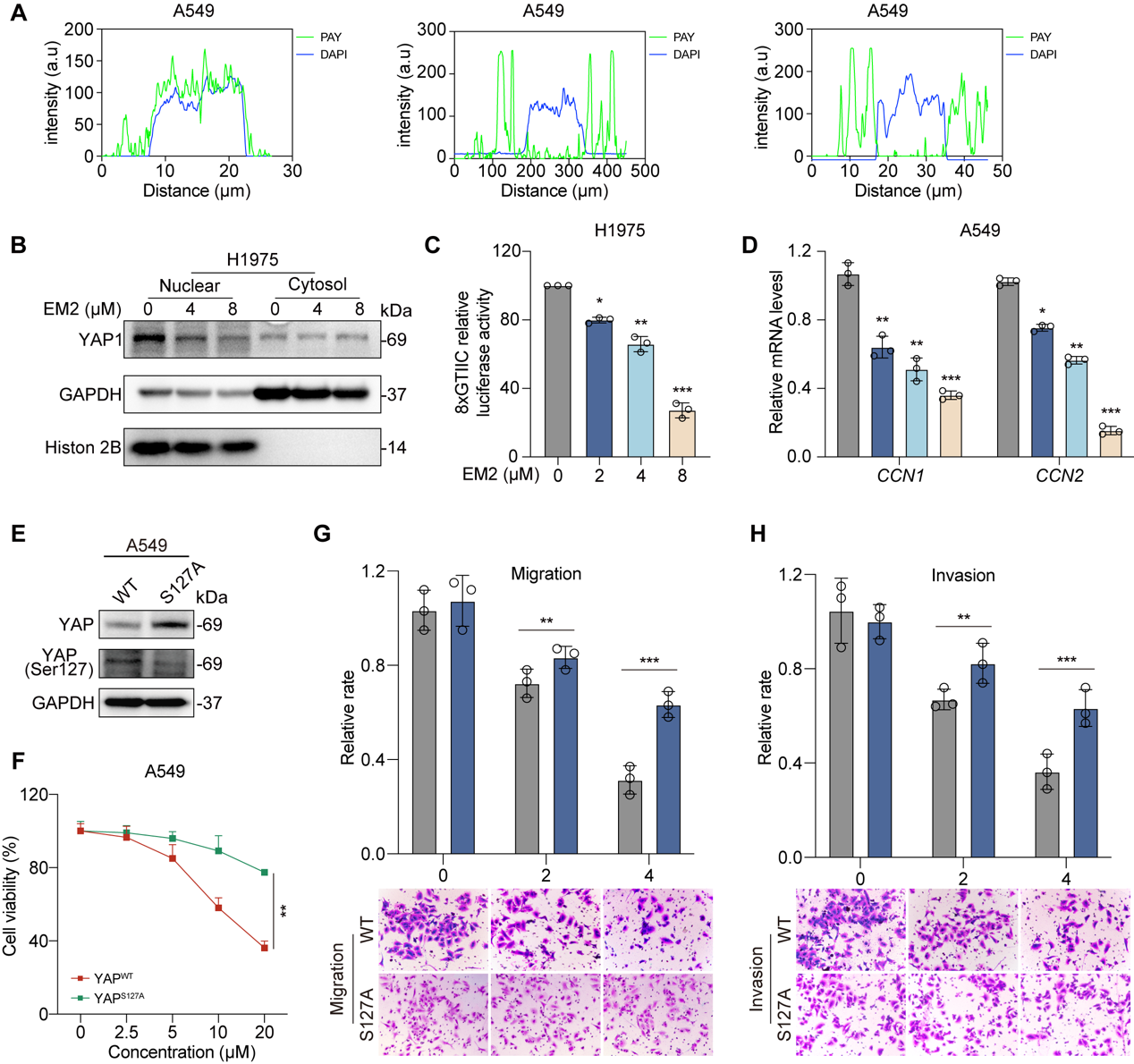


**Figure S6 EM2 exhibits anti-cell effects dependent on YAP activity.**

(A) After EM2 treatment A549, quantification of YAP localization were detected by IF staining. (B) Cytoplasmic and nuclear distributions of YAP in H1975 cells treated with or without EM2. Histone 2B and GAPDH were used as endogenous references for nuclear and cytosolic fractions, respectively. (C) Transcriptional activity of YAP/TEAD complex in H1975 cells. Cells were transfected with the reporter plasmids and treated with EM2 at the indicated concentrations for 24 h.(D) mRNA levels of CCN1 and CCN2 in A549 cells treated with EM2. RT-qPCR data were normalized to GAPDH levels and presented as fold-change compared with control cells (n = 3). (E) Western blotting was used to detect the expressions of YAP and p-YAP in YAP WT and YAP S127A A549 cells. (F) Cell viability of A549 cells transfected YAP WT and YAP S127A, treated with 0, 2.5, 5, 10, and 20 μM EM2 for 24 h. (G-H) Representative images and quantification of EM2 on the migration (G) and invasion (H) in A549 cells transfected YAP WT and YAPS127A. Following treatment with indicated concentrations of EM2, the cells were subjected to Transwell migration and invasion assays. Data are presented as mean ± SD. *p < 0.05, **p < 0.01, ***p < 0.001.


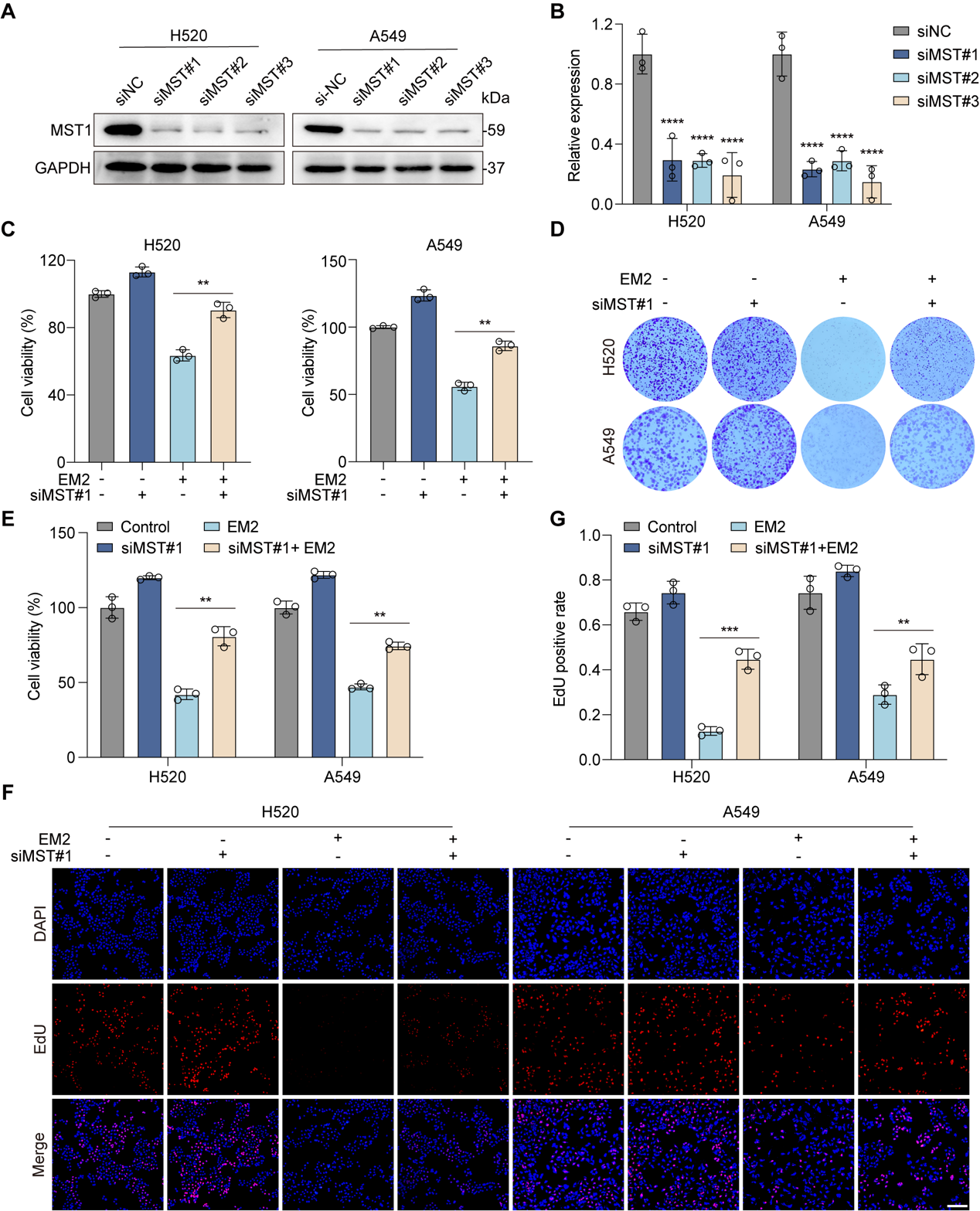


**Figure S7** The anti-tumor activity of EM2 was counteracted by MST1 knock-down in vitro.

1. B) Representative images (A) and quantification (B) of H520 and A549 cells with or without MST1 knockdown. (C) Cell viability of H520 and A549 cells with or without MST1 knockdown under EM2 treatment. (D-E) Representative images (D) and quantification (E) of colony formation assay of H520 and A549 cells with or without MST1 knockdown under EM2 treatment. (F) EdU assay of H520 and A549 cells with or without MST1 knockdown under EM2 treatment. (G) Quantification of Edu positive in H520 and A549 cells.


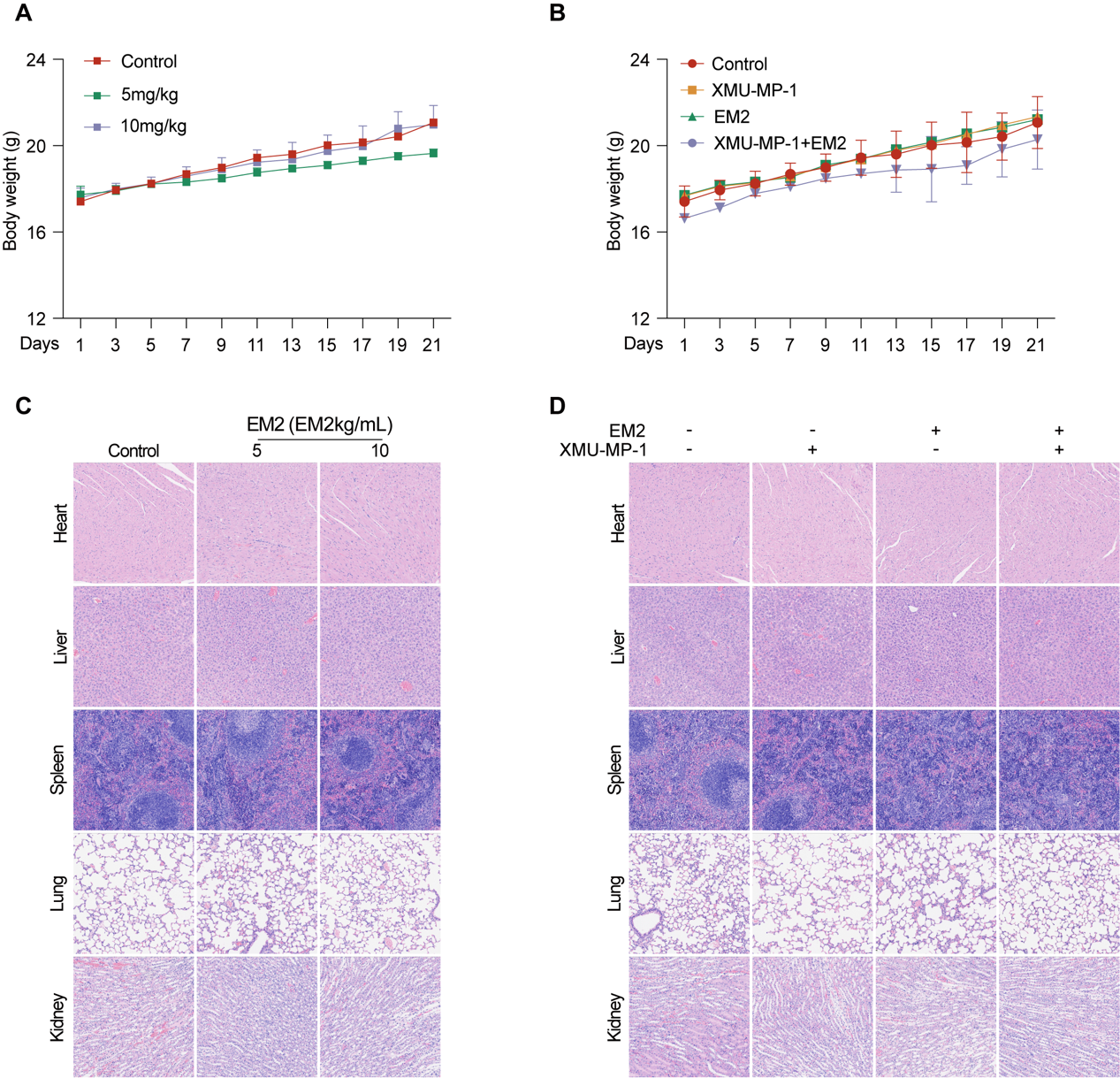


**Figure S8 EM2 demonstrated non-toxicity in mice**

(A) The body weight of mice of control group and the EM2 (5 or 10 mg/kg) group was measured every other day for those with subcutaneously transplanted tumors. (B) After subcutaneous tumor transplantation, the body weight of mice in the EM2 with or without XMU-MP-1 group was measured every other day. (C) HE staining of hearts, livers, spleens, lungs and kidneys from control and EM2 (5 or 10 mg/kg) group mice. (D) HE staining of hearts, livers, spleens, lungs and kidneys from EM2 with or without XMU-MP-1 group smice.


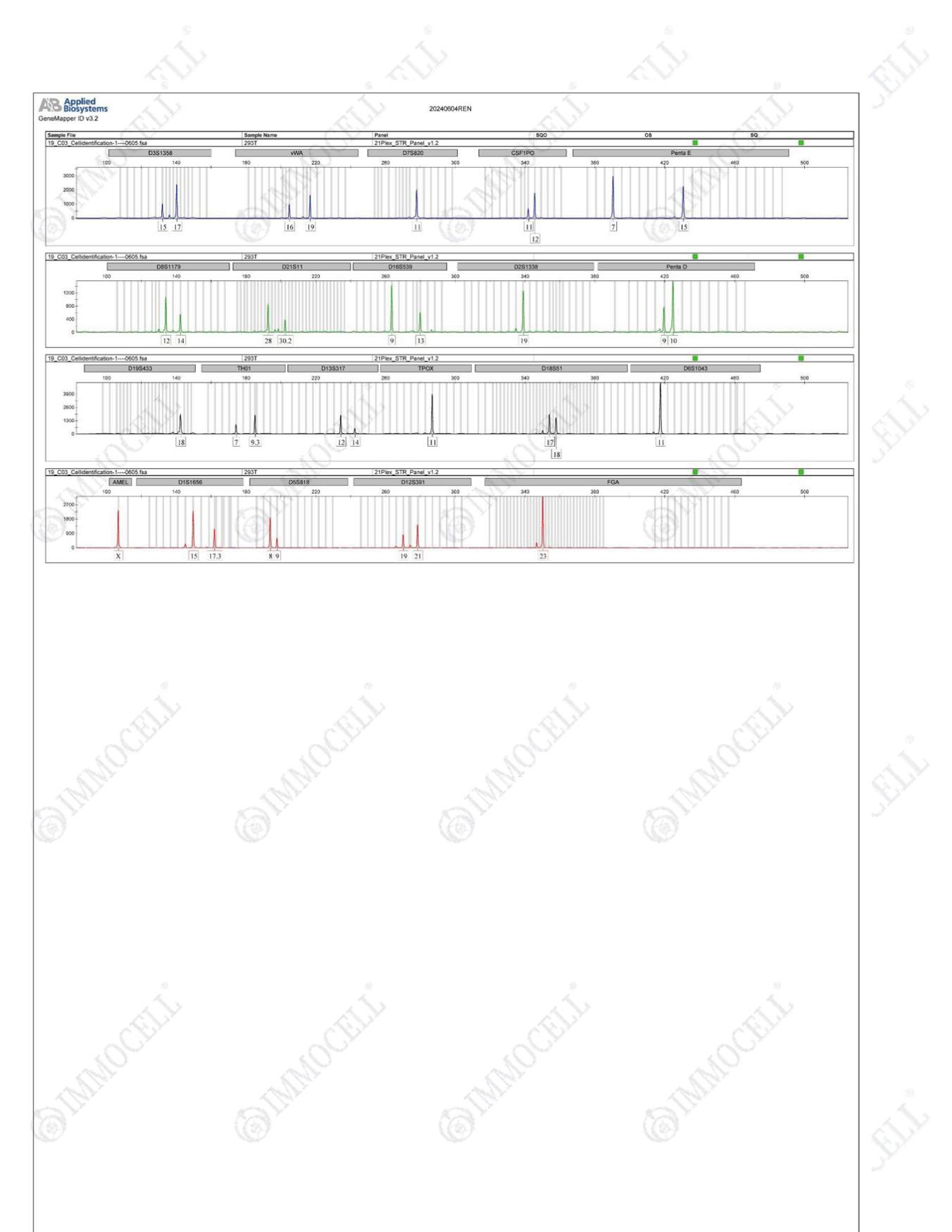


**Figure S9 Short Tandem Repeat profiling of 293T cell lines.**


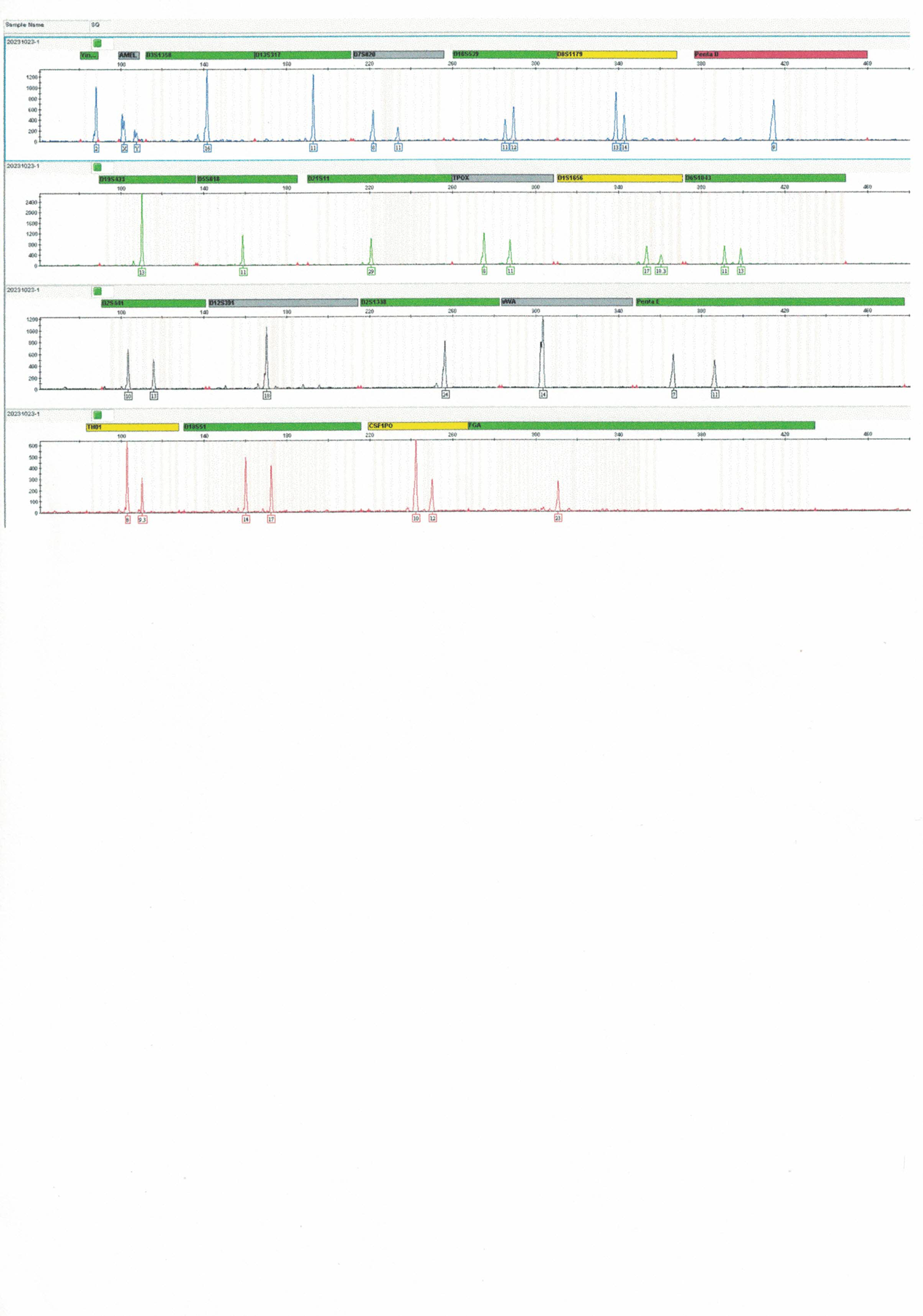


**Figure S10 Short Tandem Repeat profiling of A549 cell lines.**


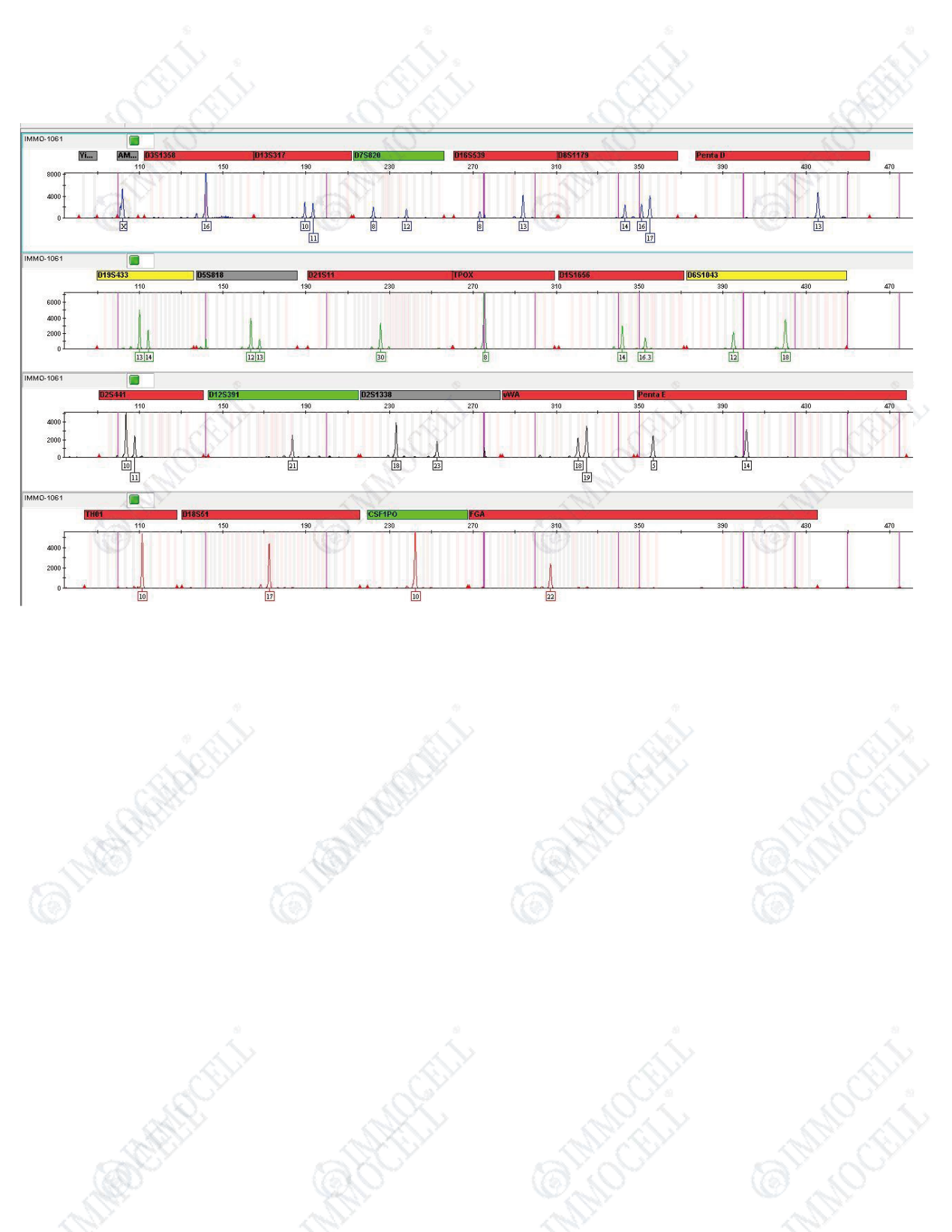


**Figure S11 Short Tandem Repeat profiling of H520 cell lines.**


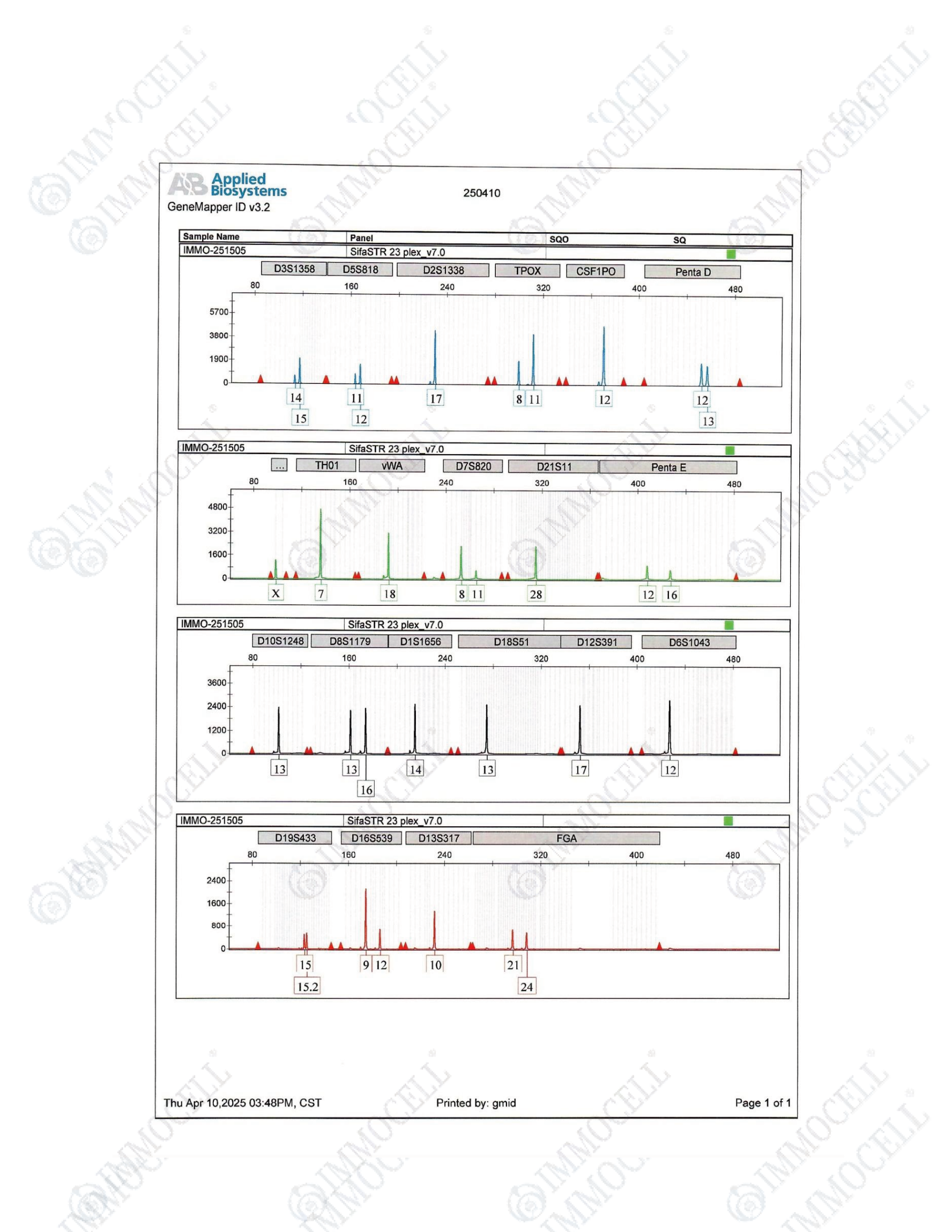


**Figure S12 Short Tandem Repeat profiling of H1975 cell lines.**


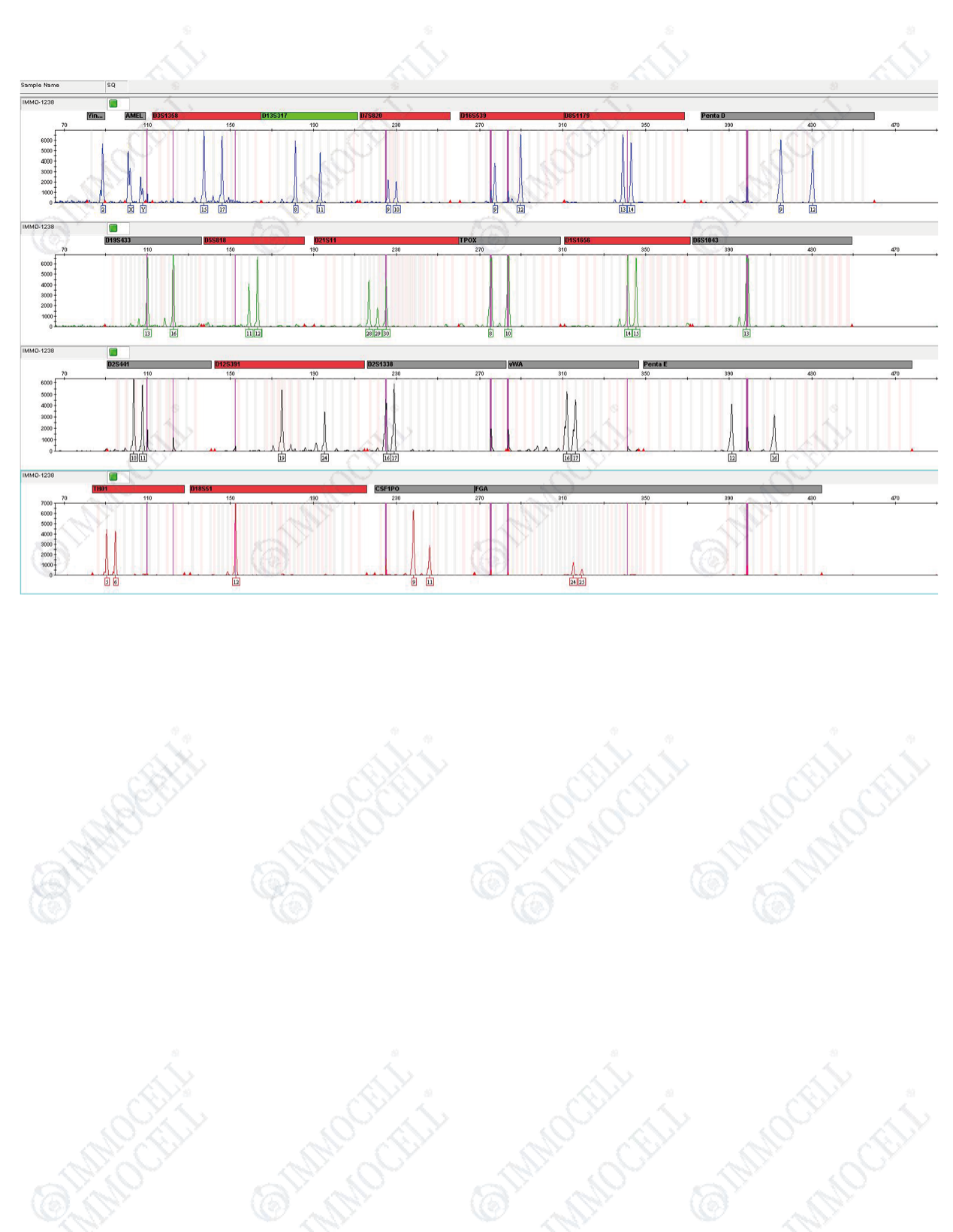


**Figure S13 Short Tandem Repeat profiling of HBE cell lines.**


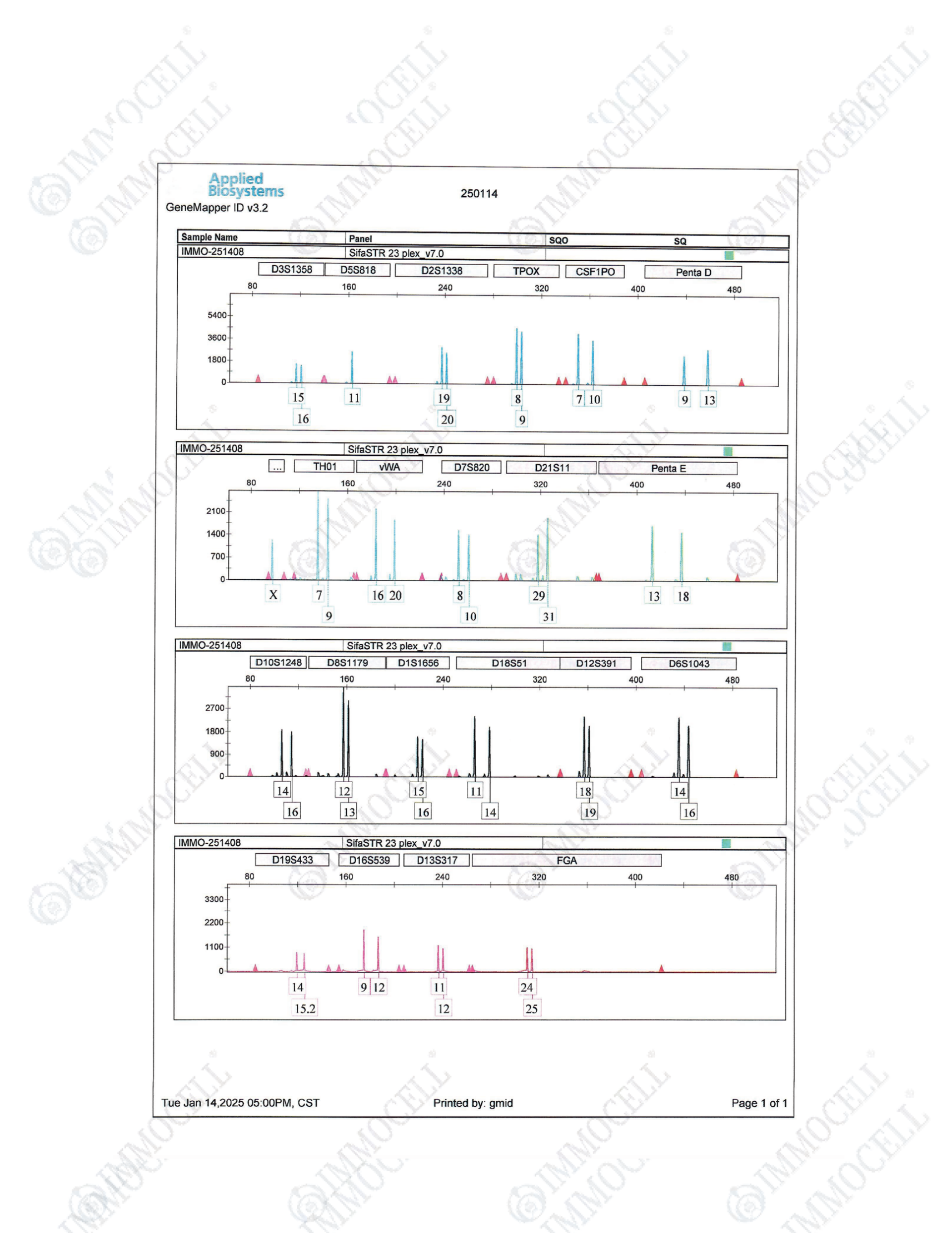


**Figure S14 Short Tandem Repeat profiling of HUVEC cell lines.**


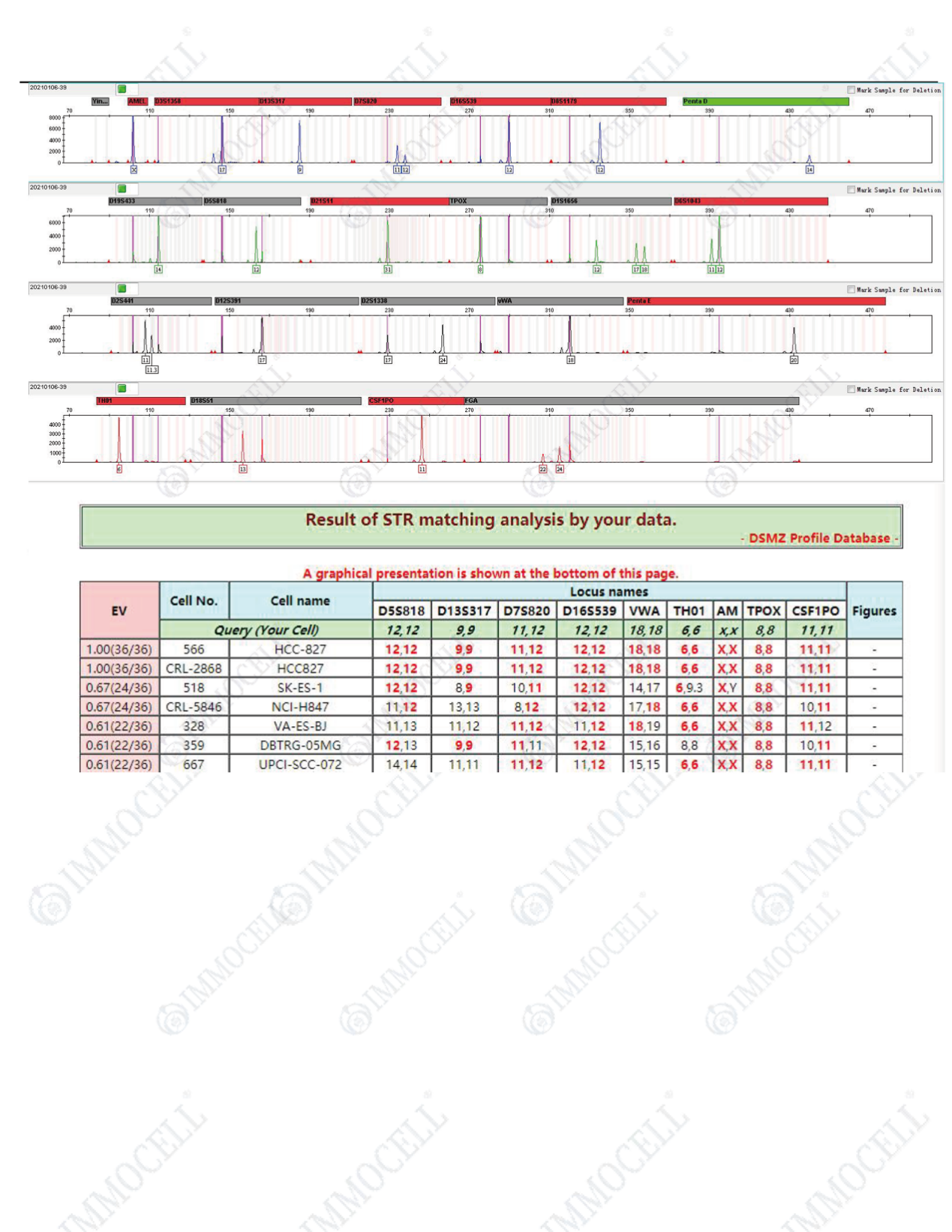


**Figure S15 Short Tandem Repeat profiling of HCC827 cell lines.**


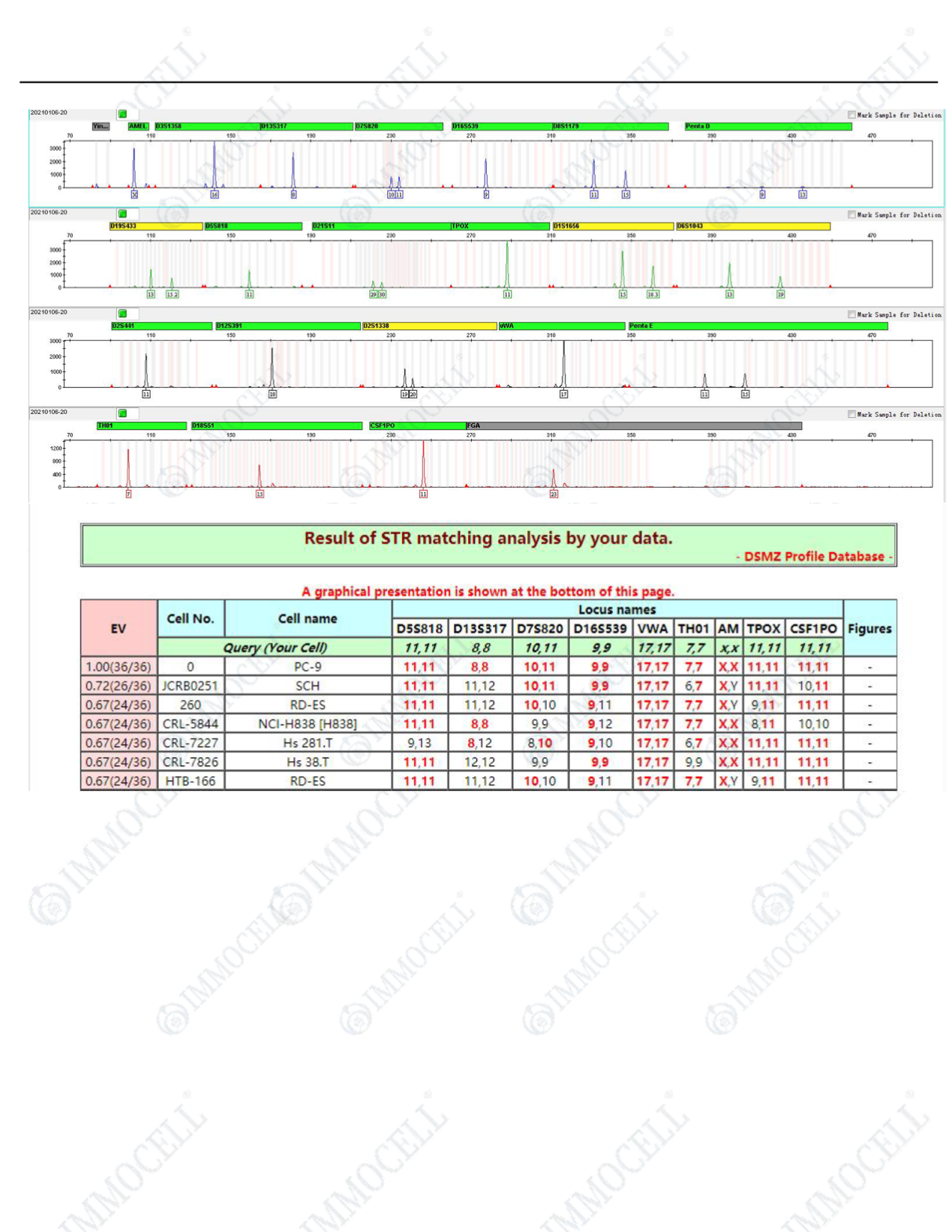


**Figure S16 Short Tandem Repeat profiling of PC-9 cell lines.**


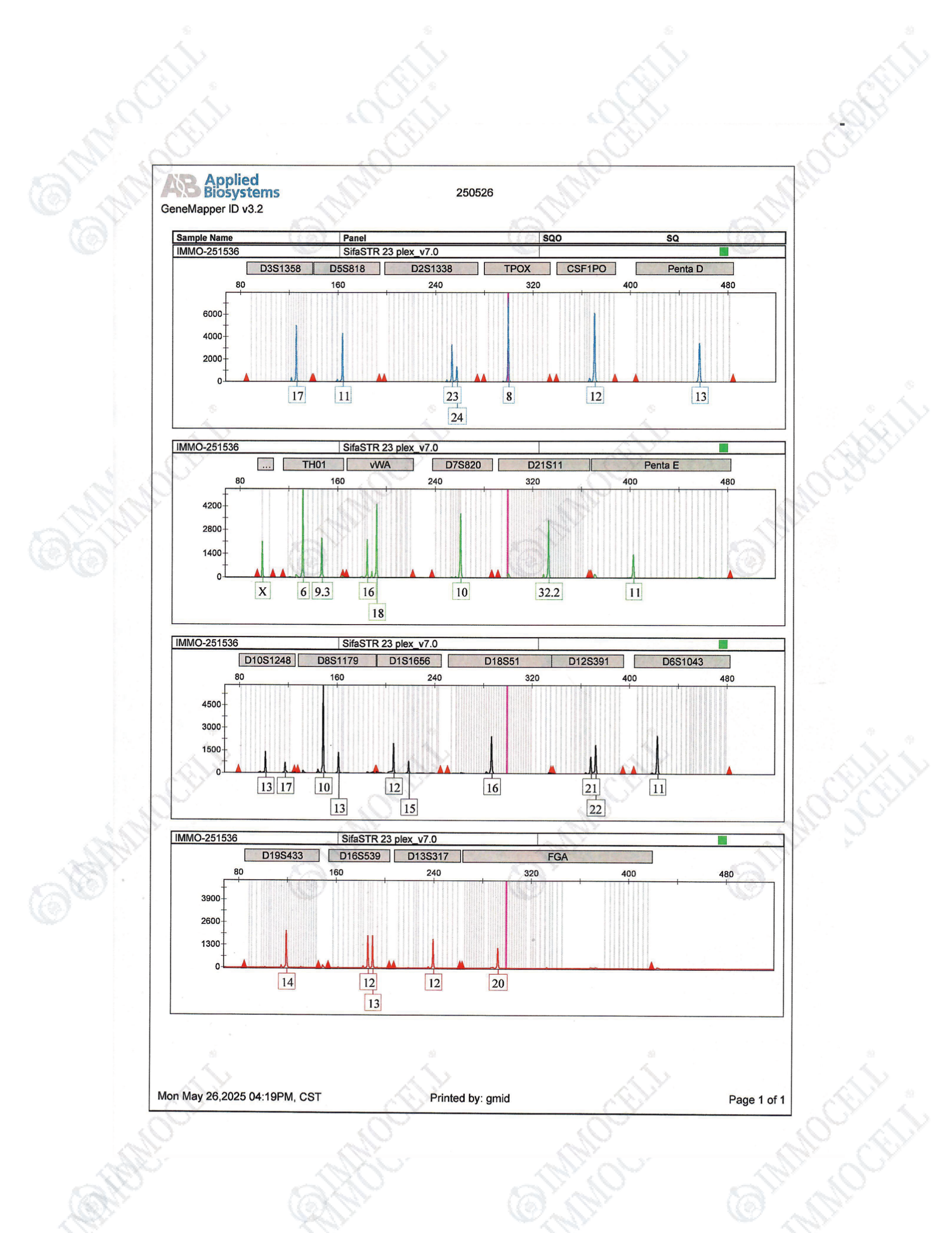


**Figure S17 Short Tandem Repeat profiling of H1299 cell lines.**
